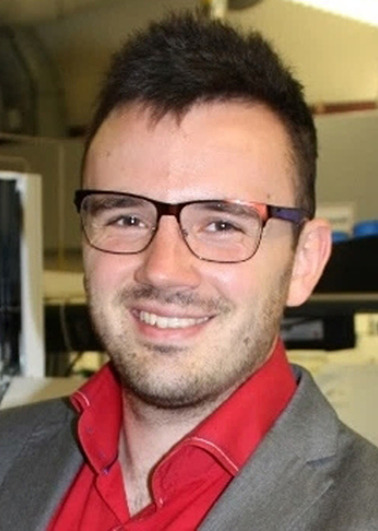# Synthetic Molecular Photoelectrochemistry: New Frontiers in Synthetic Applications, Mechanistic Insights and Scalability

**DOI:** 10.1002/anie.202107811

**Published:** 2022-02-09

**Authors:** Shangze Wu, Jaspreet Kaur, Tobias A. Karl, Xianhai Tian, Joshua P. Barham

**Affiliations:** ^1^ Universität Regensburg Fakultät für Chemie und Pharmazie 93040 Regensburg Germany

**Keywords:** electrophotocatalysis, hydrogen atom transfer, photoelectrochemistry, photoredox catalysis, preassembly

## Abstract

Synthetic photoelectrochemistry (PEC) is receiving increasing attention as a new frontier for the generation and handling of reactive intermediates. PEC permits selective single‐electron transfer (SET) reactions in a much greener way and broadens the redox window of possible transformations. Herein, the most recent contributions are reviewed, demonstrating exciting new opportunities, namely, the combination of PEC with other reactivity paradigms (hydrogen‐atom transfer, radical polar crossover, energy transfer sensitization), scalability up to multigram scale, novel selectivities in SET super‐oxidations/reductions and the importance of precomplexation to temporally enable excited radical ion catalysis.

## Graphical abstract

Synthetic photoelectrochemistry (PEC) offers new prospects for the generation and handling of reactive intermediates. PEC permits selective single‐electron transfer (SET) reactions in a much greener way and broadens the redox window of possible transformations. This Review discusses the combination of PEC with other reactivity paradigms, scalability up to multigram scale, novel selectivities in SET super‐oxidations/reductions and the importance of precomplexation.

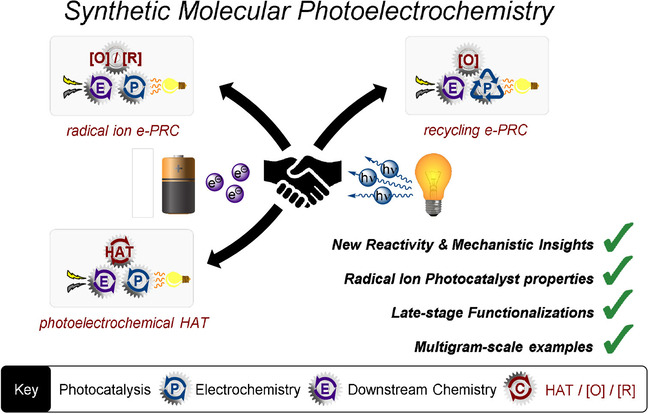

## Introduction

The field of single‐electron transfer (SET) in organic synthesis and in the late‐stage functionalization of complex molecules has expanded remarkably in the past two decades. Among this field, photoredox catalysis (PRC) and synthetic organic electrochemistry (SOE) are highly attractive to synthetic chemists due to 1) their abilities to afford reactive intermediates under mild conditions and 2) their use of light and electricity as sustainable energy sources to drive reactions.[^1^, ^2^] As powerful and generally applicable as PRC and SOE are, these chemistries present some issues on a fundamental level. PRC reactions are limited by the redox window of photocatalysts, which is in most part defined by the energy of visible photons (ca. 1.8–3.1 eV).^[3]^ UV photons that access higher energy limits come with penalties of less energy‐ and cost‐efficient reactors, safety and the direct excitation of substrate molecules leading to deleterious pathways. Visible‐light PRC reactions utilizing multiphotonic processes therefore came to the fore for overcoming the “redox energy limit”.^[4]^ To turnover PRC reactions, including these multiphotonic paradigms, large excesses of sacrificial oxidants or reductants can be required to generate active forms of the catalyst or to turnover “spent” photocatalyst. In SOE reactions, electrodes generally do not distinguish between different reaction components beyond inherent thermodynamic redox potentials of reaction components. Over‐oxidations and over‐reductions as well as solvent redox processes can plague reactions.

**Scheme 1:**
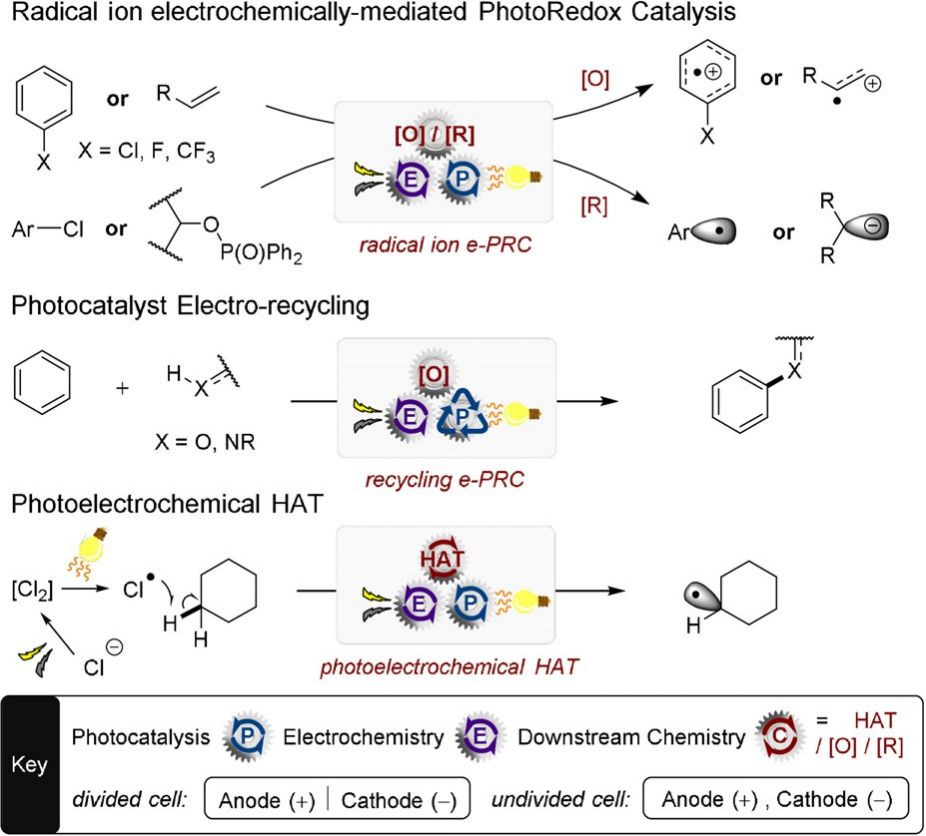
Focus of this review.

In this respect, engineering controls like tiny interelectrode distances,^[5]^ alternating potential,^[6]^ continuous flow manifolds^[7]^ or electrode surface modifications,^[8]^ have shown promise in imparting selectivity in recent years. The merger of PRC and SOE, “synthetic photoelectrochemistry”, tackles these issues and has come to the forefront of methods for SET chemistry.^[9]^ By channeling electrochemical and photochemical energy through a homogeneous (or heterogeneous) catalyst, intriguing new reactivity and selectivity opportunities are manifested. The essence of this emerging field has been captured by highlights^[10]^ as well as a full review^[9]^ and is partially covered in other reviews.^[11]^ However, the field has expanded dramatically in the last couple of years in terms of its synthetic opportunities, reactivity concepts and mechanistic understanding. In the super‐redox chemistry of electrogenerated radical ion (open‐shell) photocatalysts (Scheme 1), curious selectivities and reactivities unattainable by PRC or SOE chemistries have emerged, together with first evidences of catalyst–substrate precomplexation to rationalize otherwise time‐forbidden excited‐state reactivity. While the use of HAT as a reactivity paradigm is well established in PRC and SOE,[^12^, ^13^] its recent combination with PEC signals a broad scope of future synthetic applications. Finally, in (closed‐shell) photocatalyst electro‐recycling, methods to upscale PEC to a productive level (multigram→multigram h^−1^) are emerging, including various continuous photoflow and undivided cell batch setups.

In the following sections, we categorize the new examples as follows: 1) super‐oxidations/reductions; 2) photocatalyst electro‐recycling; 3) photoelectrochemical HAT reactions. Interfacial photoelectrochemistry^[9]^ is not covered.^[14]^ Nomenclature and abbreviations herein are as defined in our introductory review.^[9]^

## 1 Electrochemically Mediated PhotoRedox Catalysis for Selective Super‐Oxidations or Super‐Reductions

**Figure 1:**
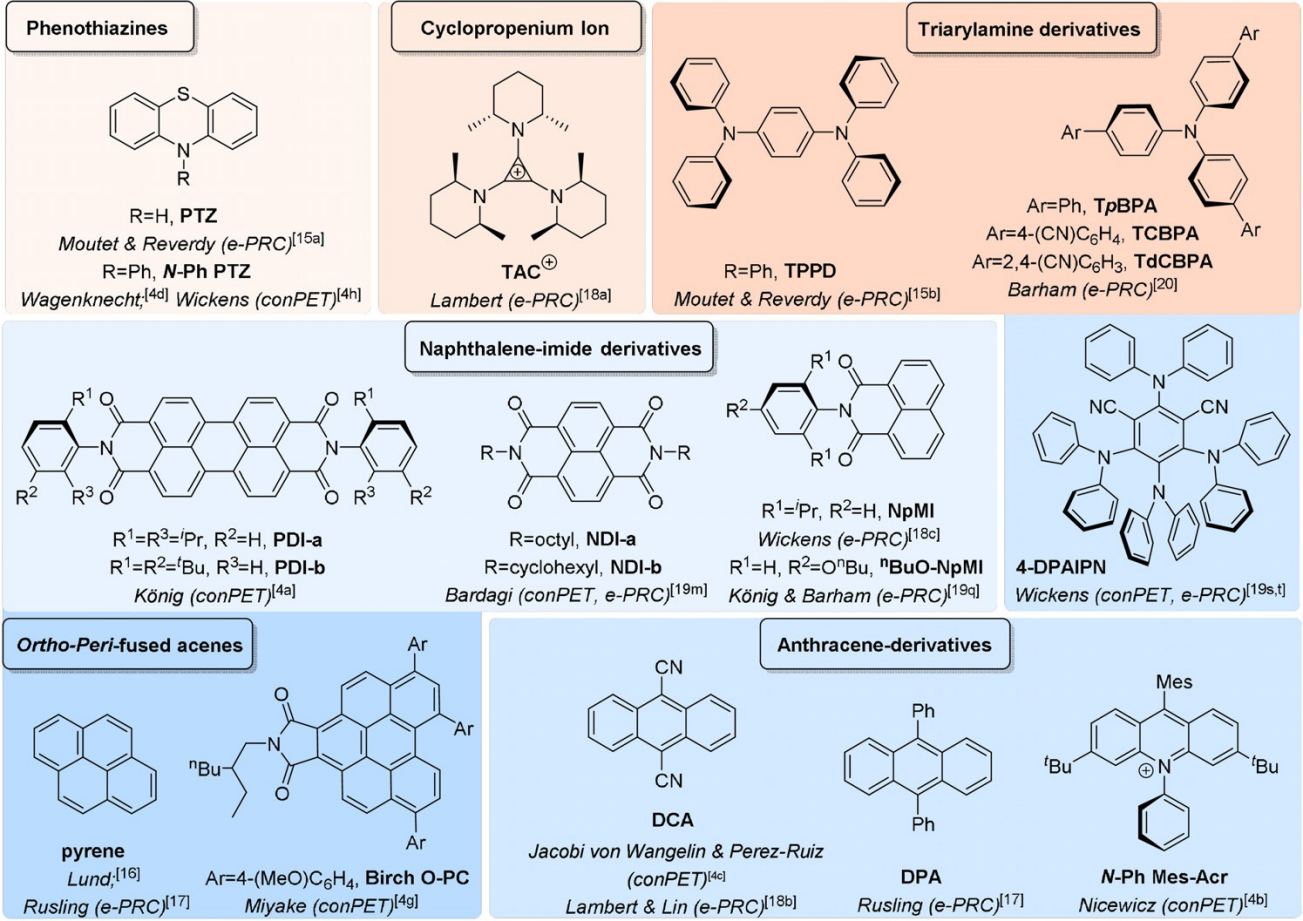
Radical ion/radical precatalysts grouped by molecular architecture: photooxidant precatalysts (red background) and photoreductant precatalysts (blue background). Shading corresponds to precatalyst family and is indicative of the oxidizing/reducing power of radical ion forms of the family but not specific cases (Table 1).

**Figure 2:**
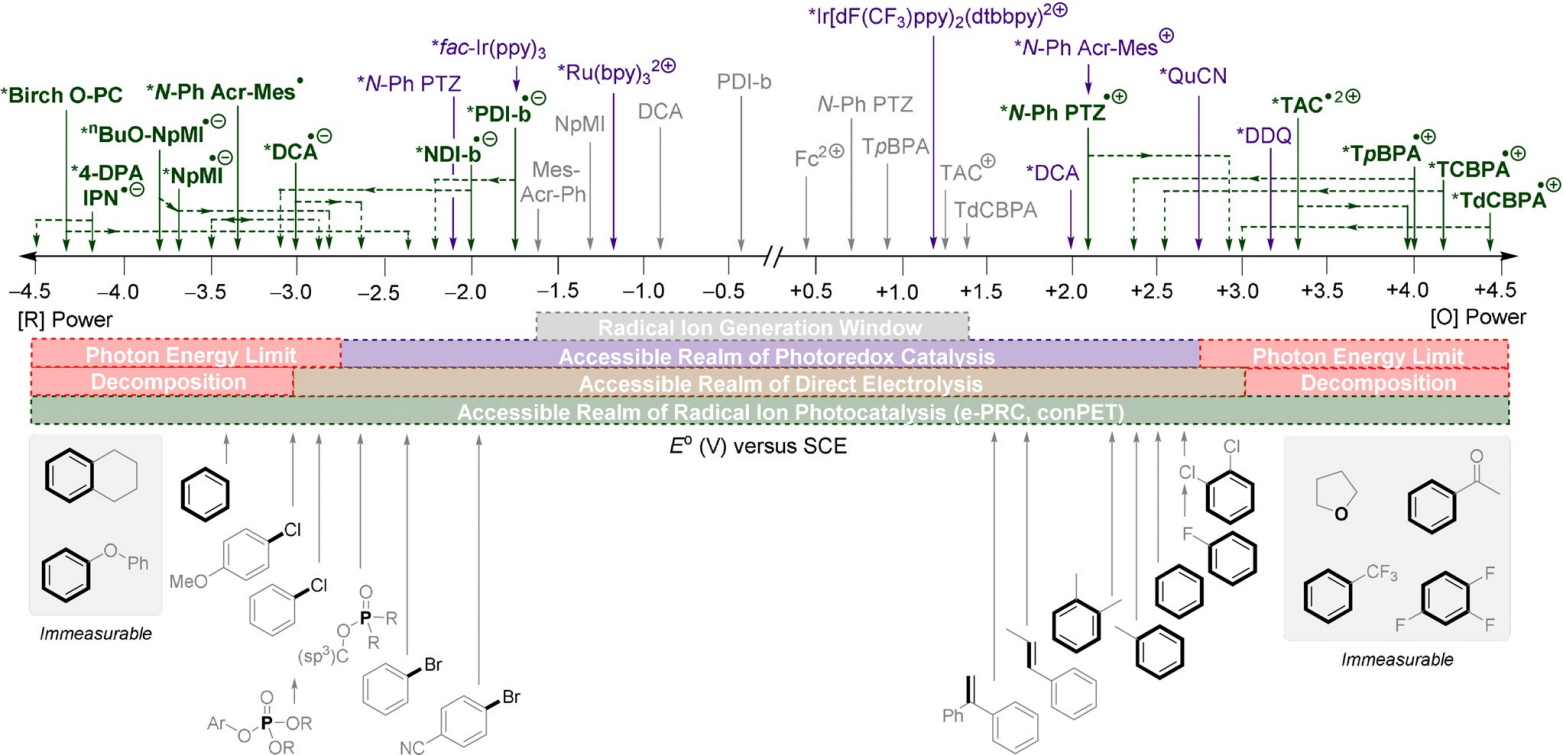
Redox benchmarking of contemporary radical ion photocatalysts compared to photoredox catalysts and redox‐challenging target substrates. Unless otherwise specified in Table 1, all catalyst redox potentials are excited‐state half‐wave potentials *E_1/2_ taken from the literature.[^2c^, ^2e^] Unless the excited‐state redox potential is explicitly claimed in the associated citation, solid lines (—) represent either the upper‐boundary excited doublet state redox potential (D_n_) and assignments are guided by the redox scope of accessible substrates. Dotted lines (‐ ‐ ‐ ‐) represent upper or lower redox bounds for the excited doublet states (D_1_ or D_n_). Substrate redox potentials taken from the literature are irreversible peak redox potentials *E* ^p/2^ [^4c^, ^18a^, ^18b^, ^19q^, ^21^, ^22^] or lie beyond the solvent redox window (>−3.0 V; >+3.0 V).^[9]^

The term “electrochemically mediated photoredox catalysis” (e‐PRC) was coined as a blanket term to describe the category of PEC involving an intimate relationship of electro‐ and photochemical steps within the same catalytic cycle, as subsequent steps.^[9]^ This broadly separates into two subcategories, “radical ion e‐PRC” and “recycling e‐PRC” (Section 2) which, although technically are mechanistically identical, are conceptually different. Radical ion e‐PRC involves electrogenerated radical ions which are photoexcitated to yield super‐oxidants or super‐reductants. The key advantage is that radical ion e‐PRC can engage compounds beyond the redox windows accessible to photoredox catalysis or synthetic electrochemistry alone (−3.0 V to +3.0 V), since photoexcited radical ion catalysts are oftentimes coloured species that possess higher redox energy (ca. ≈0.5–1.5 eV) than conventional neutral (closed‐shell) photocatalyst excited states. Seminal contributions of Moutet and Reverdy^[15]^ first demonstrated this concept in the oxidations of diphenylethene and benzyl alcohol by an electrogenerated photoexcited phenothiazine (**PTZ**) radical cation. These reports have been reviewed previously.^[9]^ Lund and Carlsson first demonstrated the reductive paradigm: dechlorination of chlorobenzene by electrogenerated photoexcited pyrene radical anions.^[16]^ Inspired by this, the reductive dechlorination of chlorobiphenyls using photoexcited 9,10‐diphenylanthracene radical anion was studied by Rusling.^[17]^ While these reports established the e‐PRC concept, their focus was not synthetic and their conditions not applicable beyond a few catalytic turnovers. The field lay dormant for several decades until its recent renaissance driven by a combination of: 1) an increasing interest in PRC and SOE,^[9]^ 2) the realization of chemically generated photoexcited radical ions as potent redox agents in synthesis[^4a^, ^4b^, ^4c^, ^4d^] and 3) recent seminal efforts in e‐PRC in organic synthesis^[18]^ as reviewed previously.^[9]^ A key platform that has underpinned rapid uptake of PRC by the synthetic community is the compilation of photocatalyst structures with their photophysical and redox properties, enabling chemists to logically plan reactions and providing best chances of success. Figure 1 depicts structures of historical and contemporary radical ion (pre)photocatalysts, while Table 1 summarizes their spectroscopic and redox properties as well as reported synthetic applications. Figure 2 summarizes redox properties of radical ion photocatalysts compared to conventional photocatalysts and example substrates. While the focus of this section is on e‐PRC, this compilation of properties should equally assist practitioners of conPET photocatalysis in planning of reactions.

**Table 1:** Properties and applications of radical ion/radical photocatalysts.

Precatalysts							Radical Ion/Radical Photocatalysts						
PRC	λ_max_ (abs)	λ_max_ (em)	τ	*E* ^0‐0^ (eV)	*E*_1/2_^[a]^ (V)	*E_1/2_^[a]^ (V)	PRC	λ_max_ (abs)	λ_max_ (em)	τ	*E* ^0‐0^ (eV)	*E_1/2_	Application
PTZ^[19a]^	<320	450	1.5–7.6 ns (S_1_)	3.20(S_1_)^[a]^	+0.60	(−2.60)	PTZ^.+[19b][b]^	834, 747, 520	–	–	1.48(D_1_)^[a]^ 2.38(D_n_)^[a]^	+2.08 +2.98	Super [O] SET: styrenes;[^4d^, ^15a^] alkylbenzenes, benzene^[4h]^
*N*‐Ph PTZ^[19c]^	<380	510, 445	0.8–2.3 ns (S_1_)	2.79(S_1_) 2.43(T_1_)	+0.68	(−2.10)	*N*‐Ph PTZ^.+[19d][c]^	864, 514	–	<36 ps (D_1_)	1.39(D_1_)^[d]^ 2.23(D_n_)^[d]^	+2.10 +2.91	
TAC^+[18a][e]^	<300	–	–	–	+1.26	–	TAC^.2+[18a],[c],[e]^	548, 497, 455	–	–	2.07(D_1_)^[d]^ 2.72(D_n_)^[f]^	+3.33 +3.98	Super [O] SET:^[18a]^ styrenes; alkylbenzenes benzene, halobenzenes dihalobenzenes
TPPD^[b]^	320^[19e]^	432^[19f]^	–	3.10(S_1_)^[19f],[g]^	+0.61^[19e]^	–	TPPD^[19e],[b]^	826, 404	–	–	1.50(D_1_)^[a]^ 3.07(D_1_)^[a]^	+2.11 +3.68	Super [O] SET:^[15b]^ benzyl alcohol
T*p*BPA^[20],[b]^	345	420	1.7 ns	3.10(S_1_)^[g]^	+0.92	(−2.18)	T*p*BPA^.+[20]^	856, 721, 419	–	4.6 ps (D_1_)	1.43(D_1_)^[a]^ 3.10(D_n_)^[f]^	+2.35 +4.02	Super [O] SET:^[20]^ alkylbenzenes, benzene, halobenzenes, dihalobenzenes, trihalobenzenes, trifluorotoluene acetophenone
TCBPA^[20],[b]^	374	435	2.2 ns	2.99(S_1_)^[g]^	+1.03	(−1.96)	TCBPA^.+[20]^	811, 695, 425	–	8.6 ps (D_1_)	1.52(D_1_)^[a]^ 3.16(D_n_)^[f]^	+2.55 +4.19	
TdCBPA^[20],[b]^	395	–	–	–	+1.34	–	TdCBPA^.+[20],[c]^	746, 639, 404	–	–	1.66(D_1_)^[a]^ 3.10(D_n_)^[f]^	+3.00 +4.41	
Pyrene^[19g],[h]^	<350	395	450 ns (T_1_)	3.14(S_1_)^[g]^ 2.00(T_1_)^[19h],[i]^	−2.10^[19h],[j]^	–	Pyrene^.−[19h],[j]^	735, 495, 385	–	–	1.69(D_1_)^[a]^ 3.22(D_n_)^[a]^	−3.79 −5.32	Super [R] SET: e^−^‐poor bromo /chloroarene C(sp^2^)‐X cleavage^[17],[19h]^
DPA ^[19h],[j]^	<410	590^[19i]^, 426^[19i]^	8.7 ns (S_1_)^[19i]^	3.09 (S_1_)^[19i],[g]^ 1.77(T_1_)^[19h],[19i],[i]^	−1.94^[19i]^	–	DPA^.−[19h],[j]^	800, 680, 610, 495	–	–	1.55(D_1_)^[a]^ 2.50(D_n_)^[a]^	−3.49 −4.44	
PDI‐a^[19j]^	526, 487	573 532	3.9 ns (S_1_)	2.35(S_1_)^[d]^ 1.20(T_1_)^[i]^	−0.43^[19k],[k],[l]^	–	PDI‐b^.−[19k],[l]^	955, 795, 700	–	≈145 ps (D_1_)	1.30(D_1_)^[a]^ 1.77(D_n_)^[a]^	−1.73 −2.20	Super [R] SET: e^−^‐poor bromo /chloroarene C(sp^2^)‐X cleavage^[19m]^
NDI‐a^[19l],[b]^	382	387	<20 ns (S_1_)^[19k]^	3.23(S_1_)^[g]^	−0.48^[19l],[l],[m]^	–	NDI‐b^.−[19k]^	755, 683, 605, 474	–	≈141 ps (D1)	1.60(D_1_)^[a]^ 2.62(D_n_)^[a]^	−2.08 −3.10	
DCA^[19n]^	415, 394, <350	435 460	14.9 ns (S_1_)	2.90(S_1_)^[g]^ 1.80(T_1_)^[19o],[i]^	−0.91	(+1.99)	DCA^.−[19p]^	708 645, 580	–^[n]^	–^[n]^	1.75(D_1_)^[a]^ 2.13(D_n_)^[a]^	−2.66 −3.04	Super [R] SET: e^−^‐neutral/rich bromo/chloro arene C(sp^2^)‐X cleavage[^18b^, ^18c^]
NpMI^[19q]^	352, 330	412	3.0 ns (S_1_)	3.27(S_1_)^[g]^	−1.32	–	NpMI^.−[19q]^	840, 745, 490, 415	535	24 ps (D_1_)^[19r],[o]^ 22 ns (ES_1_)^[p],[q]^	1.49(D_1_)^[a]^ 2.99(D_n_)^[a]^ 2.45(Q_n_)^[g,q]^	−2.81 −4.31 –	
^n^BuO‐NpMI^[19q]^	352, 330	412	3.1 ns (S_1_)	3.28(S_1_)^[g]^	−1.40	–	^n^BuO‐NpMI^.−[19q]^	840, 745, 490, 415	548	–^[o]^(D_1_) 20 ns (ES_1_) ^[p],[q]^	1.49(D_1_)^[a]^ 2.99(D_n_)^[a]^ 2.45(Q_n_)^[g,q]^	−2.89 −4.39 –	Super [R] SET: chemoselective C(sp^3^)‐O cleavages; tolerates C(sp^2^)‐X cleavages^[19q]^
4‐DPAIPN[^19s^, ^19t^]	470, 380, 350	525^[19i]^	–	–	−1.52^[r]^	(+1.12)	4‐DPAIPN^.−[19s,t]^	470, 380, 345	–	–	2.64(D_1_)^[a]^ 3.06(D_2_)^[a]^	−4.16 −4.58	Super [R] SET: e^−^‐neutral/rich chloroarene C(sp^2^)‐X cleavages; C(sp^2^)‐O and C(sp^2^)‐^+^NR_3_ cleavages[^19s^, ^19t^]
Birch O‐PC^[4g],[s]^	507, 425, 355	595	–	–	−1.23	–	Birch O‐PC^.−[4g],[t]^	715, 655, 580, 414^[c]^	580	n.d. (D_1_) 40.1 μs (ES_1_)^[p],[q]^	1.13(D_1_)^[f]^ 3.11(D_n_)^[f]^ 2.36(ES_1_)^[f,q]^	−2.43 −4.34 –	Super [R] SET: styrene, alkylbenzene Birch reductions; halostyrene dehalogenation^[4g]^
*N*‐Ph Mes‐Acr^[4b]^	420, 375	520	6.0 ns (T_1_)	2.75(S_1_)^[g]^	−1.64	(+2.15)	*N*‐Ph Mes‐Acr^.[4b],[u]^	512, 492, ≈385	–	≈100 ps (ES_1_)^[p]^	2.29(D_1_)^[f]^ 2.92(D_4_)^[f]^ 2.78(TICT)^[f,v]^	−2.87 −3.50 −3.36	Super [R] SET: e^−^‐poor/neutral /rich haloarene dehalogenations; N‐Ts cleavages^[4b]^

Unless otherwise stated, spectral data were measured in MeCN solvent and cyclic voltammetry in 0.1 M ^*n*^Bu_4_N⋅PF_6_ in MeCN as solvent, see citations for exact conditions. [a] Especially where luminescence is not reported, an estimation was made for *E* ^0‐0^ by taking the photon energy corresponding to λ^abs^_max_ for both the lowest and highest energy transition and *E_1/2_ was calculated by the simplified Rehm–Weller equation. [b] In DCM. [c] Spectral properties were determined from in situ electrogenerated species. [d] The red edge of the UV‐vis absorption band was taken for *E* ^0‐0^. [e] Spectral data were obtained under the preparative reaction conditions. [f] Taken from the calculated TD‐DFT vertical transition that corresponded most closely to the excitation wavelength in the preparative reactions. [g] *E* ^0‐0^ was determined by the intersection of absorption (most red‐shifted) and emission (most blue‐shifted) spectral bands. [h] In cyclohexane. [i] *E* ^0‐0^ was determined by another or unspecified means, see the cited study. [j] In DMSO. [k] For **PDI‐b**. [l] In DMF. [m] **NDI‐a** and **NDI‐b** have the same redox potentials. [n] Controversy exists over the reported luminescence/lifetimes of **DCA^.−^** which likely derive from other species (10‐cyanoanthrolate anion).^[19p]^ [o] Transient absorption spectroscopy of a direct analog (2,4‐disubstituted) of **NpMI^.−^** reportedly afforded rapid photobleaching of the sample in 0.1 M ^*n*^Bu_4_N⋅PF_6_ in DMF and the lifetime could not be determined;^[19k]^ however, the lifetime of **NpMI^.−^** itself was successfully determined in a recent study in 0.1 M ^*n*^Bu_4_N⋅PF_6_ in DMAc.^[19r]^ [p] This study^[19q]^ did not assign the measured lifetime to a specific excited state and the excited state was thus denoted “ES_1_”. [q] A quartet state was tentatively proposed as a candidate for the long‐lived species. Since this state was found to be catalytically inactive in the PET step due to no quenching of the excited state in the presence of substrate, an excited state potential is not given. [r] In 0.1 M ^*n*^Bu_4_N⋅PF_6_ in DMF. [s] Spectral data measured in CHCl_3_ and cyclic voltammetry in 0.1 M ^*n*^Bu_4_N⋅PF_6_ in DMAc. [t] In THF. [u] In hexane. [v] The reactive excited state was proposed to be a twisted intramolecular charge transfer state (TICT).

**Figure 3:**
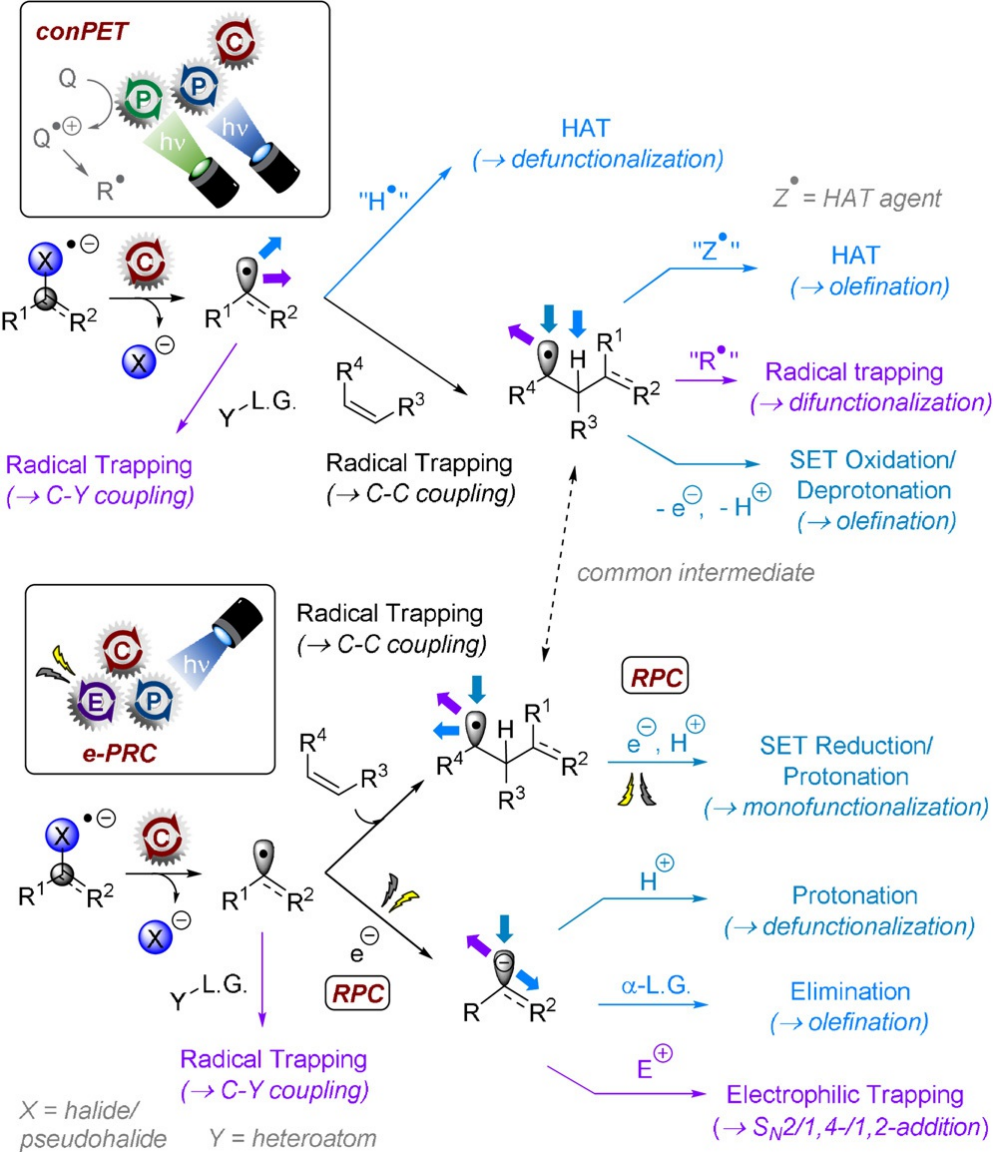
Downstream chemistry reactivity paradigms of conPET vs. e‐PRC. RPC: radical polar crossover.

**Comparison of conPET and e‐PRC**: While conPET and e‐PRC methods are analogous in their active catalyst excited state and their scope of SET reactions, their downstream chemistry is fundamentally distinct and complementary. The reactivity paradigms are compared in the context of super‐reductions (Figure 3). In both cases, following SET from the photoexcited radical ion catalyst, C–X bond cleavage affords a radical intermediate. In conPET, this radical intermediate, typically an aryl C(sp^2^) radical, can undergo 1) hydrogen‐atom transfer (with solvent or with by‐products derived from sacrificial reductants in the radical ion catalyst generation step), 2) addition to a heteroatom trapping agent or 3) addition to an unsaturated partner (usually an electron‐rich aromatic) in a C−C coupling reaction. In the case of (3), the subsequent radical either reacts with a HAT agent (“Z**^.^**” → olefination) or is oxidized (by “Q**^.^** ^+^”) and deprotonated, in both cases reforming the unsaturation. The HAT agent (“Z**^.^**”) or oxidant (“Q**^.^** ^+^”) is the by‐product from the sacrificial electron donor that was required to form the radical anion photocatalyst. For example, the α‐amino radical or *N*‐radical cation of a trialkylamine electron donor.

In e‐PRC, different fates await the radical intermediate following initial SET and C–X bond cleavage, by virtue of the absence of sacrificial chemical reductants and their by‐products. The radical can undergo the same radical trapping reactions as with conPET, but instead of HAT with solvent its electroreduction to a carbanion occurs in a radical polar crossover (RPC) fashion. The resulting carbanion intermediate can undergo 1) protonation, 2) elimination of an α‐leaving group or 3) electrophilic trapping. The radical intermediate following C−C coupling can undergo further reduction and subsequent protonation, or can engage in radical trapping depending on applied potential. Thereby, e‐PRC offers the unique advantage of a user‐potentiocontrollable mechanism.

While radical ion photocatalysts are a common theme in conPET and e‐PRC chemistries, the focus of this review now turns to recent synthetic applications of the latter. Elegant, synthetic conPET applications are reviewed elsewhere.[^2e^, ^4i^, ^4j^, ^4k^, ^11b^]

**C(sp^2^)−N bond formations**: In the oxidative direction, e‐PRC has witnessed impressive synthetic applications in C(sp^2^)−N bond formations. The Buchwald–Hartwig coupling is an important, widespread reaction for forging C(sp^2^)−N bonds, but relies on prefunctionalization of the C(sp^2^)‐containing arene (an aryl halide or pseudohalide) for oxidative addition of a Pd catalyst. A powerful alternative is the direct C(sp^2^)–H activation of arenes by SET oxidation to their radical cations, which undergo S_N_Ar‐type attack by *N*‐containing nucleophiles. The Nicewicz group first demonstrated this concept using PRC with the Fukuzumi catalyst, an acridinium salt (**Mes‐Acr^+^**) with a high excited‐state oxidation potential (*E_1/2_=+1.88 V vs. SCE). Electron‐rich arenes such as anisole underwent direct C(sp^2^)−N bond formations with azoles.

Elegant work by the Lambert group exemplified the power of e‐PRC to breach the upper redox limit of single‐photon PRC^[18a]^ which has been reviewed elsewhere.^[9]^ They disclosed a trisaminocyclopropenium cation (**TAC** ^+^) as an electroactivated photoredox catalyst (e‐PRCat) whose excited state could oxidize benzene, chlorobenzene and up to dichlorobenzenes as arene partners. Trifluorotoluene gave no product, defining the upper limit of the excited state's oxidative power. While its scope of applications was broad, the exotic structural architecture of the catalyst does not seem amenable to modifications. Structural modification of a photoredox catalyst core to a family of derivatives with different photophysical and redox properties is a concept underpinning much of the success of field of PRC with transition metal or organic photocatalysts.[^2c^, ^2e^]

**Figure 4:**
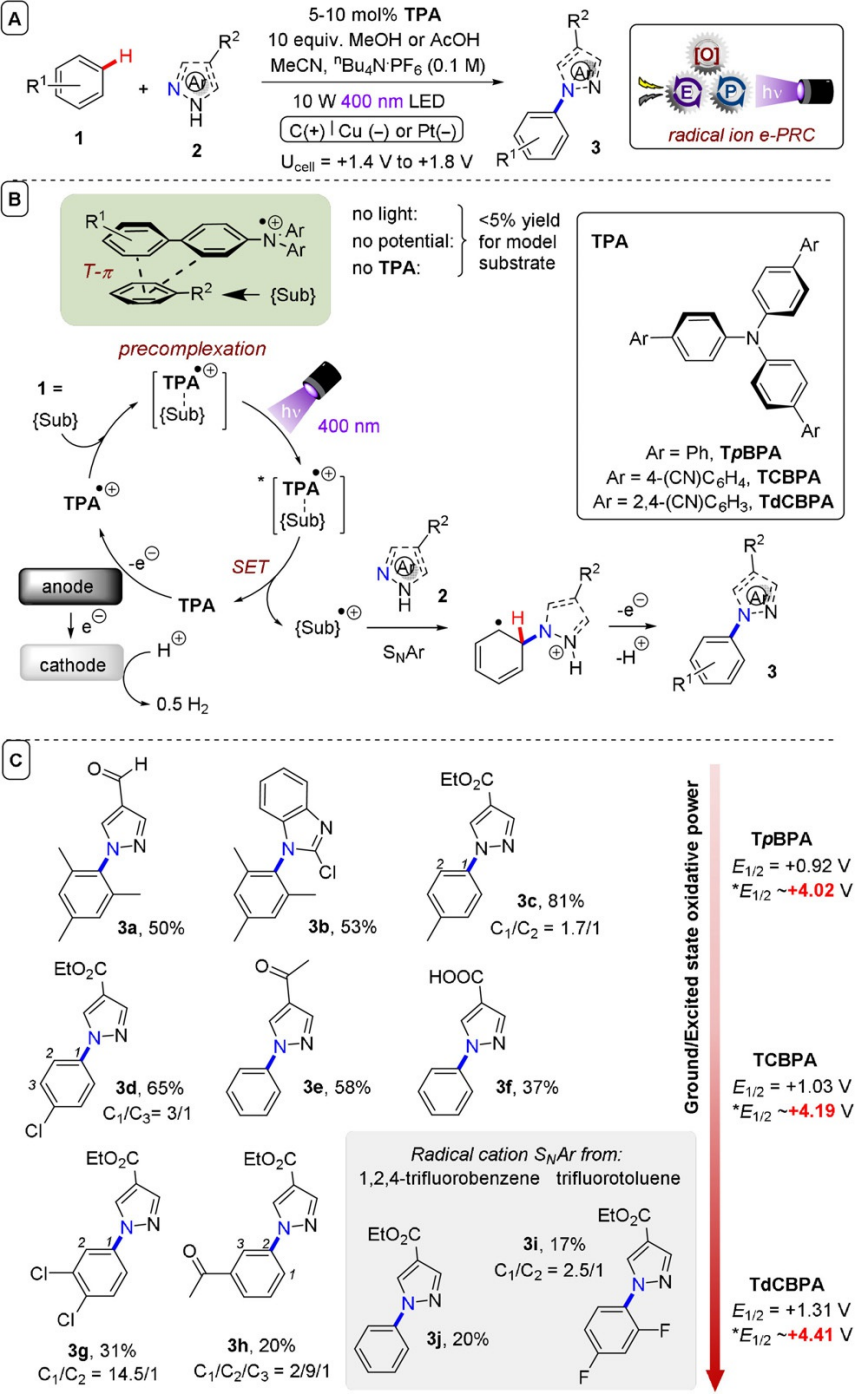
A) **TPA**s as a library of tunable superoxidative photocatalysts. B) Proposed precomplexation mechanism and **TPA** catalysts used in this study. C) Selected examples.

In the context of e‐PRC, the Barham group disclosed tri(*p*‐substituted) arylamines (**TPA**s) as a tunable class of e‐PRCat for super‐oxidations (Figure 4 A).^[20]^ The **TPA**s are easily accessed and customized in a single step by Suzuki or Ullmann coupling reactions. A ubiquitous structural motif in hole‐transport functional materials (OLEDs, photovoltaics)^[23]^ and as mediators in electrolysis,^[24]^ the photophysical and redox properties of **TPA**s and their bench‐stable (isolable) radical cations^[25]^ have a rich history of characterization^[26]^ that precedes even that of ruthenium polypyridyl complexes.^[27]^ In their e‐PRC reactions, **TPA**s firstly undergo anodic oxidation to their corresponding strongly coloured radical cations at a constant cell potential of +1.4 to 1.8 V (Figure 4 B). Altering *para*‐substituents on the **TPA** allows facile access to radical cation photocatalysts with different oxidative powers. Use of a moderate‐power **TPA** (**T*p*BPA**) allowed C(sp^2^)–H amination of alkylbenzenes with high selectivity; aldehyde‐bearing pyrazoles and arene benzylic positions were tolerated without oxidation. Introduction of a *para*‐cyano group on the peripheral aromatic ring gave a higher‐power **TPA** (**TCBPA**) which allowed C−H amination of benzene and chlorobenzene in good yields and tolerated free carboxylic acids known to undergo decarboxylation in PRC^[28]^ and Kolbe oxidation pathways in SOE.^[29]^ Using the most powerful **TPA** (**TdCBPA**), the oxidative SET C−H aminations of dichlorobenzenes, fluorobenzene and even acetophenone were achieved. SET activations of very electron‐poor trifluorobenzene and trifluorotoluene were achieved, leading to S_N_Ar reactivity (Figure 4 C). The utility of triarylamines as a family of e‐PRCats that could be tuned to target substrates was successfully demonstrated.

A key mechanistic question comes to mind when discussing the photochemistry of excited radical ions (doublet states), given that these species are well known to exhibit ultrashort (<1 ns) lifetimes that prohibit their diffusion‐controlled photochemistry. It had been tentatively hypothesized in previous studies involving conPET reduction that a precomplexation of the substrate and radical ion photocatalyst occurs to rationalize otherwise temporally forbidden photochemistry.[^4a^, ^4g^] A key advantage of **TPA**s as electroactivated photocatalysts is that they can be easily oxidized and isolated as their bench‐stable radical cations.[^25c^, ^25d^] This allowed the authors to measure *TPA^.^ ^+^ lifetimes as short as <10 ps by transient absorption spectroscopy, clearly ruling out diffusion‐controlled photochemistry. Precomplexation between **TPA^.^** ^+^s and arene substrates was proposed as the key to overcoming ultrashort (ps) lifetimes (Figure 4 B). It was postulated that steric effects in the precomplex formation rationalized both 1) the reactivity trend of xylene and dichlorobenzene isomers, since preparative reaction yields increased in the order: 1,4‐ <1,2‐disubstituted arenes; and 2) the lack of reactivity of iodobenzene. The increasing order of *E* ^p^_ox_: 1,4‐ <1,2‐disubstituted arenes^[18a]^ and the highly accessible redox potential of iodobenzene meant that such behaviour was unexpected and contra‐thermodynamic.

**Figure 5:**
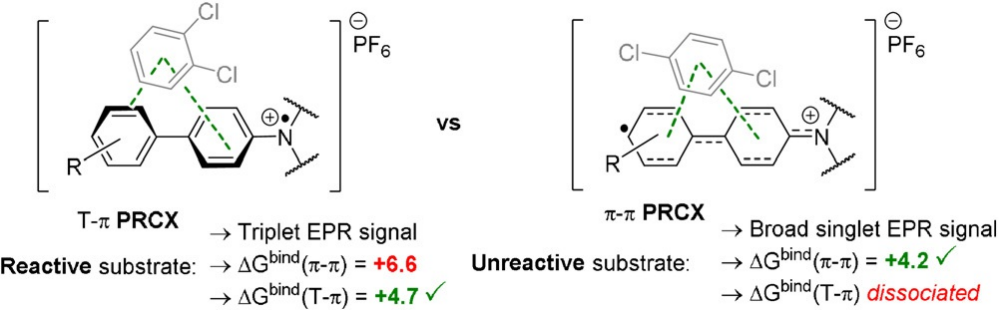
Candidate precomplexes of reactive (left) vs. unreactive (right) arenes.

**Figure 6:**
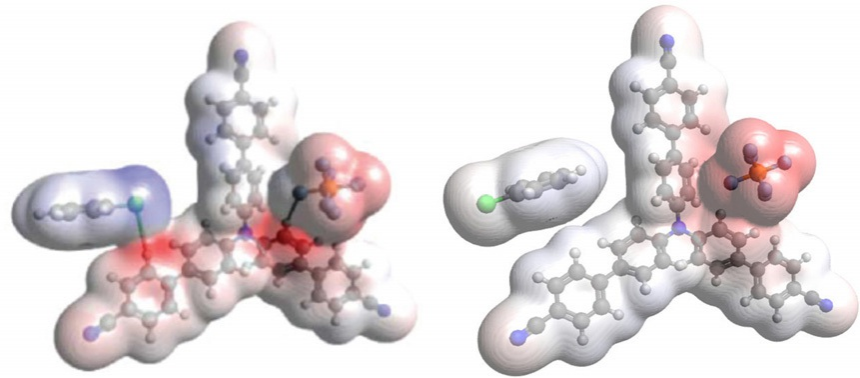
Calculated spin densities of T–π precomplexes of **TCBPA^.^** ^+^ with chlorobenzene where the halogen faces “in” (left) or “out” (right).

**Figure 7:**
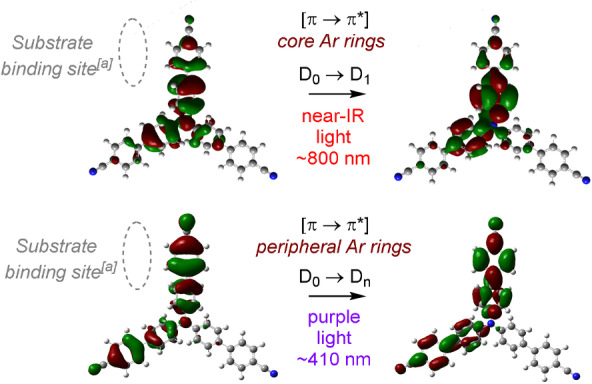
Orbital transitions for photoexcitation of **TCBPA^.^** ^+^ corresponding to D_0_→D_1_/D_n_. [a] Binding site predicted by DFT calculations.

The presence of precomplexes was identified spectroscopically via changes in the UV‐vis spectra and EPR spectra of isolated **TPA^.^** ^+^s in the presence of arene substrates. The most striking comparison was when 1,2‐ or 1,4‐dichlorobenzene substrates were added to **TCBPA^.^** ^+^. The former “reactive” substrate perturbed the EPR signal to a “triplet shape”, confirming spin density localization on the N atom in the precomplex. The latter “unreactive” substrate perturbed the signal to a “broad singlet”, indicating another type of precomplex with spin density delocalization away from the N atom, which may stabilize the radical cation and decrease its excited state reactivity to SET. DFT calculations (ωB97X‐D or B3LYP functionals) found optimized structures involving T–π or π–π interactions. Relative binding energies revealed “reactive” substrates favoured T–π geometries and “unreactive” substrates favoured π–π geometries (Figure 5). In chlorobenzene's precomplex with **TCBPA^.^** ^+^, the calculated spin density changed when the Cl atom faced “in” but did not change when facing “out”, providing a clue about the preferred geometry of precomplexes involving unsymmetrical haloarenes (Figure 6).^[30]^ Although **TPA^.^** ^+^s absorb strongly in the visible region (ca. 600–900 nm) corresponding to their first (doubly degenerate) excited states (D_0_→D_1_), only shorter wavelengths (400 nm) gave reactivity, suggesting anti‐Kasha photochemistry. The ability to access higher‐order doublet photoexcited states of **TPA^.^** ^+^s with 400 nm provides as high as *E ^p^_ox_=+4.4 V vs. SCE as an upper boundary,^[31]^ and would rationalize the oxidation of arenes so electron poor that they exceed the measurable solvent window of cyclic voltammetry. TD‐DFT calculations, which agreed well with UV‐vis spectra of **TPA^.^** ^+^s,^[32]^ revealed the near‐IR transition (D_0_→D_1_) involved a π–π* transition localized at the core aromatic rings (Figure 7). In contrast, the transition closest to ≈400 nm (D_0_→D_n_) involved a π–π* transition at the peripheral aromatic rings. This is precisely the binding location of substrate arenes as found by geometry optimizations. Precomplexation may rationalize synthetically productive anti‐Kasha photochemistry by the localization of hole density at the binding site of the substrate, priming the precomplex for ultrafast SET (faster than internal conversion; D_n_→D_1_).

While the simple computational model of precomplexes is consistent with experimental observations, deeper investigations with other DFT theory levels,^[33]^ different (non‐DFT)^[34]^ theories and different precomplex candidates^[35]^ are warranted for a more holistic picture. Nonetheless, this study evidenced how radical ion–substrate precomplexes not only circumvent ultrashort lifetimes of doublet excited states, but even circumvent internal conversion, allowing a greater proportion of the photon energy to be harnessed. Moreover, the study indicated how radical ion–substrate precomplexes give rise to different selectivities than SET chemistry involving diffusion‐controlled SOE or PRC.

**Figure 8:**
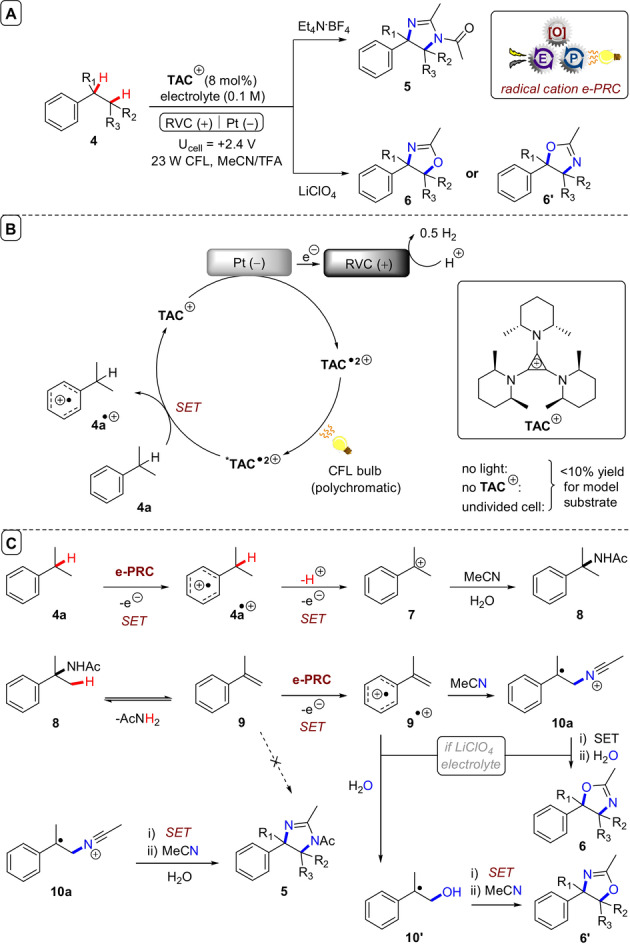
A) e‐PRC vicinal diamination of alkylarenes. B) Catalytic cycle. C) Proposed downstream chemistry mechanism.

**C(sp^3^)−N bond formations**: In an impressive synthetic application of e‐PRC, the Lambert group used **TAC** ^+^ for vicinal diamination of alkylarenes (**4**, Figure 8 A).^[36]^ Depending on the electrolyte, alkylated arenes undergo vicinal diamination to afford either 3,4‐dihydroimidazoles (**5**) or oxazoline derivatives (**6** and **6′**). In the anodic chamber of a divided cell, **TAC** ^+^ is oxidized to its coloured dication radical **TAC** ^.2+^. Upon photoexcitation, the highly oxidative *TAC ^.2+^ (*E_1/2_=+3.33 V vs. SCE) engages alkylarenes like cumene **4 a** in SET (Figure 8 B). Upon loss of a proton from **4 a** ^.+^ and SET oxidation, benzylic carbenium ion **7** is generated and nucleophilic attack of MeCN (as solvent) leads to Ritter‐type amidation (Figure 8 C). The resulting acetamide (**8**) undergoes acid‐catalyzed elimination to α‐methylstyrene (**9**). Subsequent e‐PRC oxidation of **9** by *TAC ^.2+^ affords radical cation **9^.^** ^+^ and MeCN solvent adds. Further oxidation and addition of a third equivalent of MeCN was proposed to afford dihydroimidazole **5** in a Ritter‐type fashion.^[37]^ Control reactions using α‐methylstyrene (**9**) that led to polymerization seemed to contradict this proposal. However, an equilibrium between **8** and **9** would generate small quantities of **9** in situ, mitigating polymerization. LiClO_4_ electrolyte presumably alters the stability of cationic intermediates and the addition of H_2_O to **9^.^** ^+^ or **10 a** affords **6′** and **6**, respectively.

**Figure 9:**
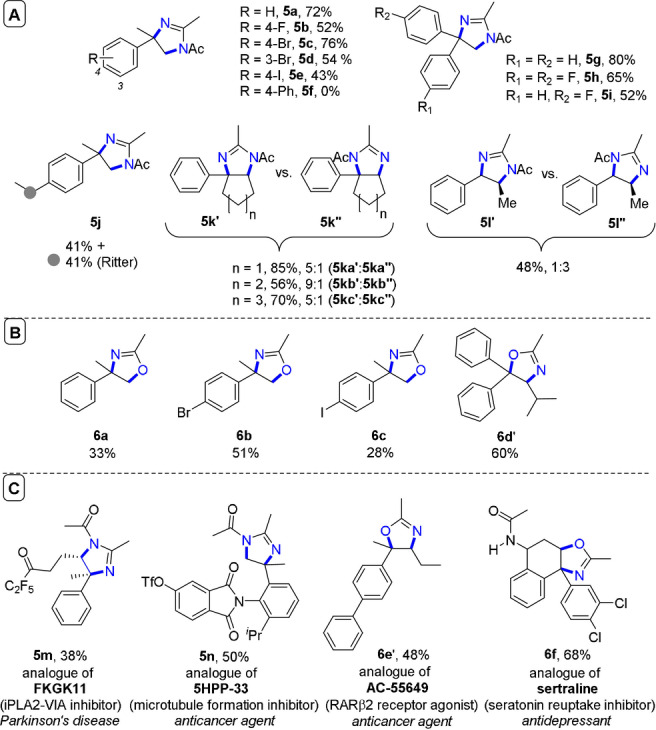
A) e‐PRC vicinal diamination substrate scope of dihydroimidazoles. B) Substrate scope of oxazolines from using LiClO_4_ electrolyte. C) Late‐stage functionalizations of bioactive molecules.

When applied to secondary alkylbenzenes, halide substitution was well‐tolerated, while alkyl *para*‐substitution on the arenes led to formation of Ritter‐type benzylic by‐products (**5 j**, Figure 9 A). Cyclic systems were successfully functionalized, with preference for 4‐phenylimidazoles over 5‐phenylimidazoles. In primary alkylbenzenes, the regioselectivity was inverted. Most impressively, the simple change of electrolyte from Et_4_N**⋅**BF_4_ to LiClO_4_ diverted the mechanism to form oxazolidines in an overall oxyamination. The methodology was amenable to late‐stage functionalizations of pharmaceutical compound analogues (Figure 9 C). Either dihydroimidazole or subsequent 1,2‐diamine scaffolds could be accessed by a simple modification of the workup procedure, while β‐amino alcohols derived from oxazolidine hydrolysis, signaling a broad and industrially relevant scope of future applications for this chemistry.

In the context of C−H aminations, it is important to mention that **DDQ** as a neutral (closed‐shell) photocatalyst^[38]^ can achieve C(sp^2^)–H aminations of electron‐deficient arenes up to dichlorobenzenes, either under PRC^[39]^ with a co‐oxidant or under recycling e‐PRC (see Section 2).^[40]^ However, larger, substoichiometric quantities (10–20 mol %) are typically required and the evolution of hydrogen cyanide upon contact of this catalyst with moisture presents safety concerns.^[41]^ **DDQ** is a rather potent ground‐state oxidant that can react with *N*‐nucleophiles,^[39]^ while **TAC^+^** and **TPA** catalysts do not suffer such issues.

**Figure 10:**
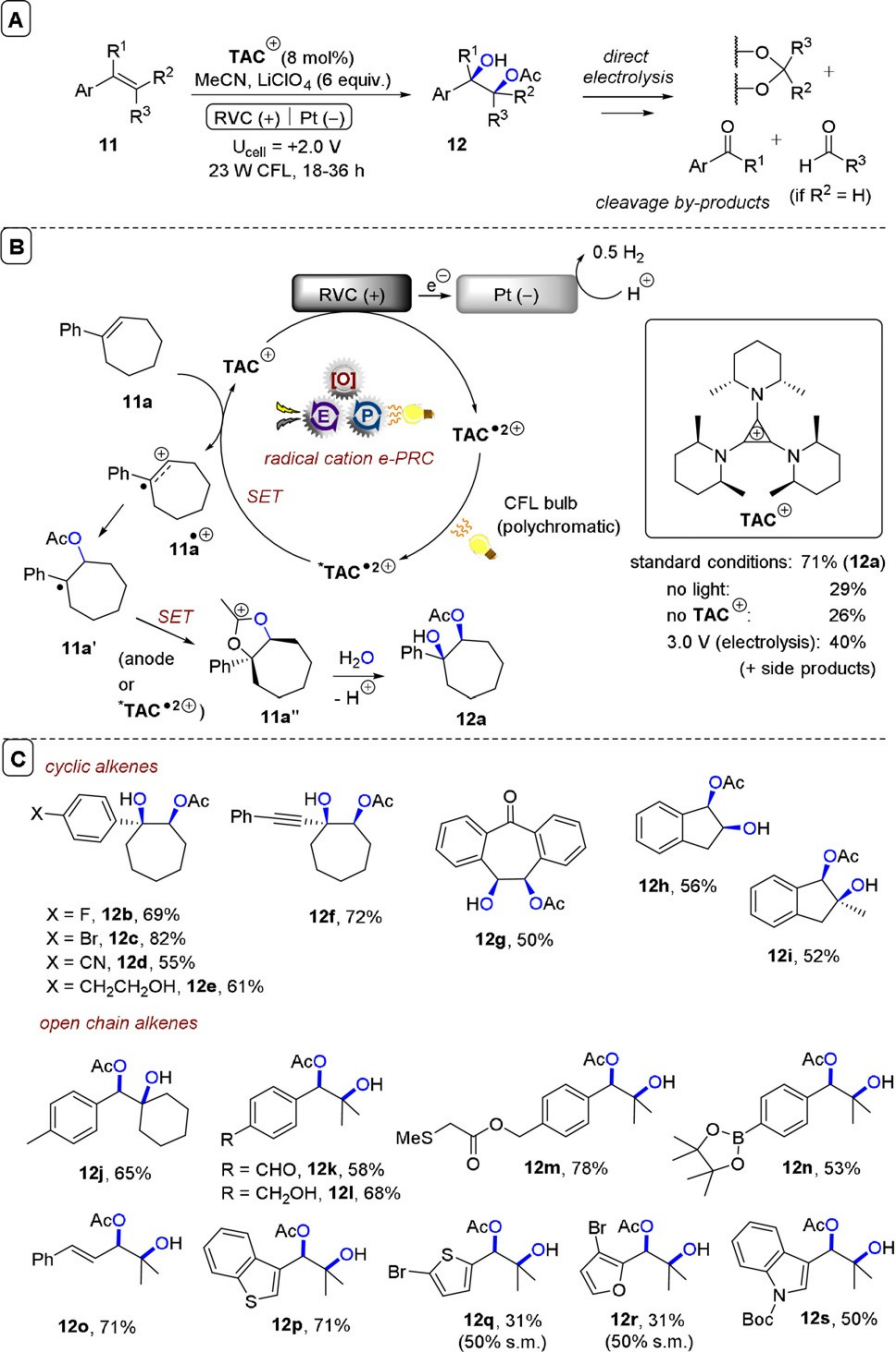
A) e‐PRC acetoxyhydroxylation of aryl olefins. B) Proposed mechanism. C) Substrate scope of dihydroxylations, simple substrates.

**Figure 11:**
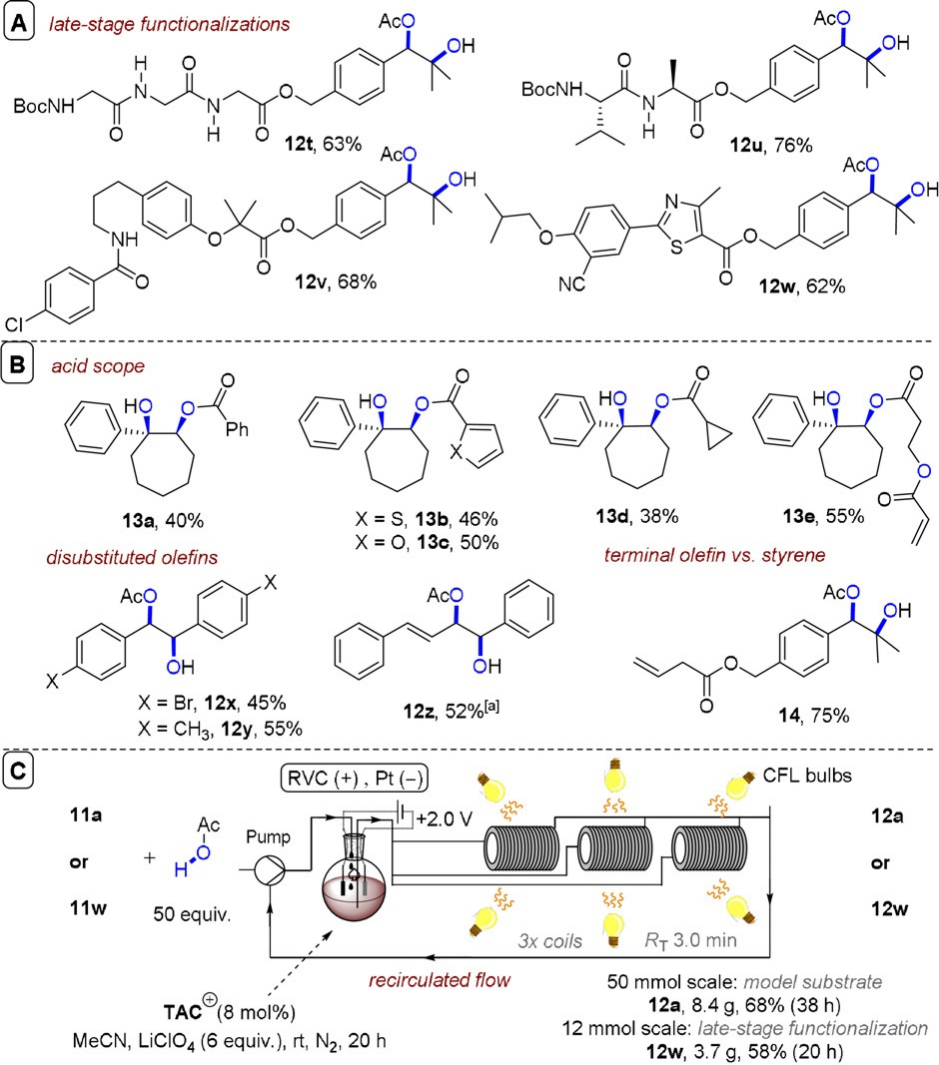
A) Late‐stage acetoxyhydroxylation. B) Substrate scope of acid partners and disubstituted olefins showing terminal olefin tolerance. C) Recirculated flow recycling e‐PRC acetoxyhydroxylation of the model substrate or a complex molecule.

**C−O bond formations**: The Lambert group demonstrated the acetoxyhydroxylation of aryl olefins (**11**) by radical ion e‐PRC using **TAC** ^+^. Transition‐metal‐catalyzed dioxygenation reactions of olefins are well‐known, but the authors noted the need for transition‐metal‐free approaches to obviate the expense and toxicity of certain metals. While electrochemical approaches using “cation pool” strategies are attractive,^[42]^ in dioxygenations they oftentimes lead to the cleavage of deoxygenated products to carbonyl‐ and acetal‐derived by‐products (Figure 10 A).^[43]^ Direct electrolysis employing DMSO and DMF as nucleophiles to attack electrogenerated olefin radical cations has successfully afforded dihydroxylated olefins.^[44]^ The Lambert group's use of radical ion e‐PRC and acetic acid led instead to acetoxyhydroxylations, providing a mild platform to access products such as **12 a** in 71 % yield. Direct electrolysis likely occurs to an extent under the relatively high potential of +2.0 V; control reactions without light or **TAC** ^+^ did afford product **12 a** but in low yields. Direct electrolysis at +3.0 V increased the yield of **12 a** to 40 % but led to over‐oxidation and cleavage to aldehyde, ketone and acetal by‐products. In the mechanism, **11 a** is oxidized by *TAC^.^ ^2+^ to its radical cation, which undergoes nucleophilic attack by AcOH to afford benzylic radical **11 a’** (Figure 10 B). Oxidation of **11 a’**, either by anodic potential or by *TAC^.^ ^2+^, induces intramolecular cyclization to **11 a”**, primed for attack by H_2_O to afford **12 a**. Cyclic olefins were acetoxyhydroxylated to give **12 b**–**12 i** in good to excellent (50–82 %) yields. A striking feature is the selectivity of this method compared to prior chemical and electrolytic reports. Benzylic methyl groups, alcohols and aldehydes were all tolerated (**12 j**–**12 l**). A free alkylsulfide, a Bpin ester, a product‐bearing styrene and electron‐rich heterocycles were all tolerated in the syntheses of **12 m**–**12 s** (31–78 % yields). Yields of furan‐ and thiophene‐containing **12 q** and **12 r** were low (31 %), but primarily due to lack of conversion. Selectivity was further exemplified in the late‐stage acetoxyhydroxylations of various arylolefin‐conjugated amino acids and complex molecules (Figure 11 A). Other acids were tolerated, affording **13 a**–**13 e** in modest to good (38–55 %) yields (Figure 11 B). Interestingly, regioselectivity was inverted in these cases. Acrylic acid as a nucleophile underwent oligomerization in the synthesis of **13 e**, which may be rationalized by Kolbe‐type oxidation of acrylic acid and radical addition,^[45]^ yet authors deemed this unlikely in the absence of base. Finally, multigram amounts of products were achieved via a recirculated flow setup (Figure 11 C). Electro‐activation of **TAC** ^+^ was done in a batch undivided cell and the reaction mixture was recirculated through three CFL‐irradiated coils with a total residence time (*R*_T_) of 3 min, affording up to 8.4 g of **12 a** and 3.7 g of **12 w** without appreciable yield losses after 20–36 h.

**Figure 12:**
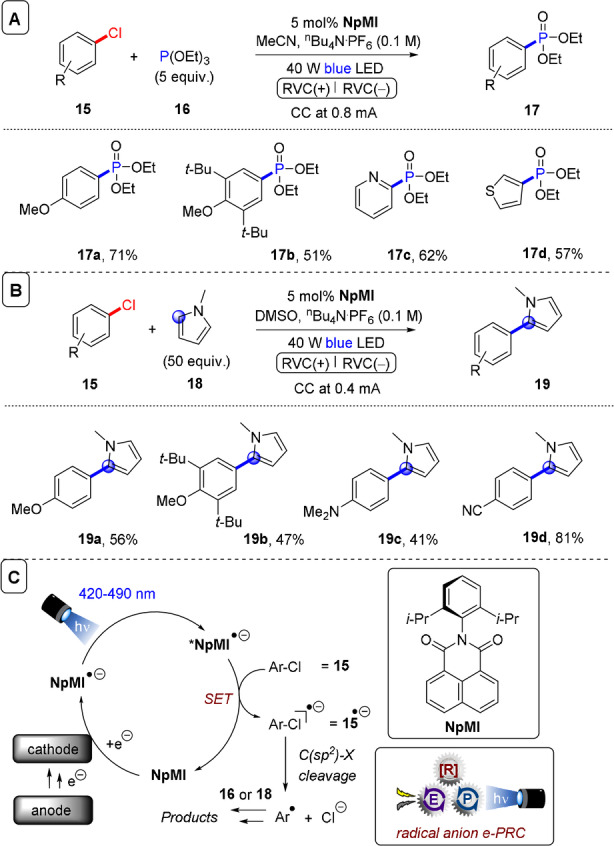
A) e‐PRC reduction of aryl chlorides and trapping of aryl radicals, substrate scope with triethyl phosphite. B) Substrate scope of trapping with *N*‐methylpyrrole. C) Catalytic cycle.

**C(sp^2^)–X bond cleavages**: On the reduction side, Lambert and Lin disclosed 9,10‐dicyanoanthracene (**DCA**) as an electroactivated photoreductant for super‐reductions of aryl halides,^[18b]^ which was previously reviewed.^[9]^ Simultaneously, the Wickens group reported an *N*‐arylmaleimide (**NpMI**) as a catalyst that achieves the same transformation (Figure 12).^[18c]^ Inspiration was drawn from seminal work of the König group on perylene diimide (**PDI‐b**) photocatalysts, which are known to form stable radical anions (**PDI‐b^.^** ^−^) that can be photoexcited to reduce electron‐poor aryl halides in a consecutive photoelectron transfer (conPET) mechanism.^[4a]^ e‐PRC reactions with **DCA** and **NpMI** catalysts extended the scope of aryl halide partners to electron‐neutral and electron‐rich, and conditions provide substantial advantages for such challenging SET reductions over traditional Birch‐type conditions which are practically undesirable (dissolving alkali metals in liquid ammonia).

**Figure 13:**
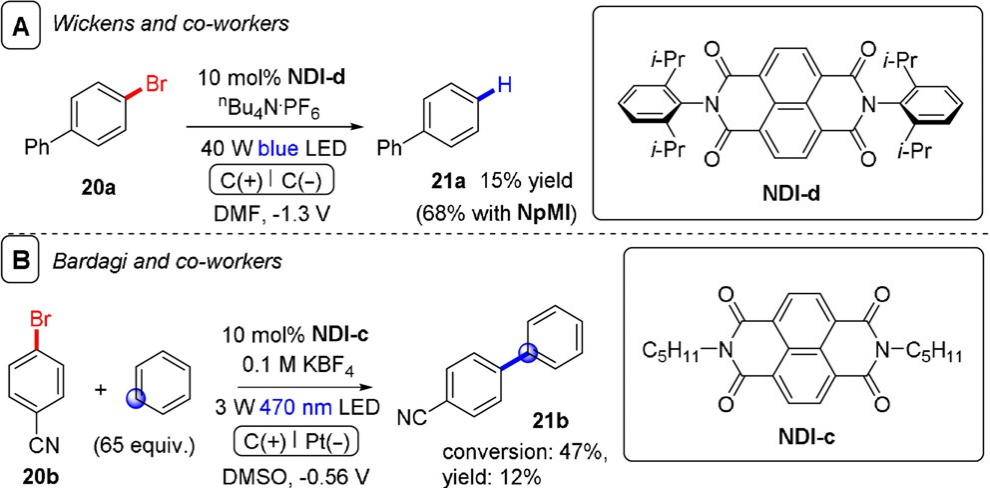
A) e‐PRC dehalogenation of 4‐bromobenzonitrile using a naphthalene diimide precatalyst. B) e‐PRC reduction of 4‐bromo‐1,1′‐biphenyl using a naphthalene diimide precatalyst and trapping with benzene.

Due to the Beer–Lambert relationship, in e‐PRC SET takes place in bulk solution where the electrogenerated catalysts is irradiated, not at the electrode surface. This leads to immediate deactivation of the photoexcited state; thus e‐PRC exhibits a key benefit in harnessing the reactivity of aryl(sp^2^) radicals. Direct electrolytic reduction of aryl halides that takes place at the electrode surface suffers the issue that aryl(sp^2^) radicals are thermodynamically easier to reduce than their aryl halide precursors, leading inevitably to overall dehalogenation.^[21a]^ Wickens and co‐workers found that diimide catalyst architectures (**PDI** and naphthalene diimide, **NDI**) were ineffective catalysts for the reduction of electron‐neutral aryl halides,^[18c]^ likely because the high stabilization of the radical anion prohibits super‐reductive chemistry in its photoexcited state. In their optimized e‐PRC reaction conditions, the new catalyst *N*‐(2,6‐diisopropylphenyl)naphthalene monoimide (**NpMI**) is first reduced to its coloured radical anion by a cathodic constant current of 0.8 mA. Upon photoexcitation with blue LEDs, photoexcited radical anion *NpMI^.^ ^−^ engages aryl chlorides as challenging as 4‐chloroanisole (**15 a**) in SET reduction to aryl radicals (Figure 12 C). Aryl(sp^2^) radicals either underwent overall dehalogenation, or were trapped with triethylphosphite (Figure 12 A) or *N*‐methylpyrrole (Figure 12 B) to afford products **17** and **19**. Notably higher preparative yields were obtained compared to direct electrolysis (which gave noticeable decomposition), demonstrating the key selectivity benefit of radical ion e‐PRC. When the debromination of 4‐bromobiphenyl (*E* ^p^_red_≈−2.43 V vs. SCE)^[46]^ was used to optimize reaction conditions, a bis‐*N*‐(2,2′,6,6′‐diisopropyl)naphthalene diimide (**NDI‐d**) precatalyst afforded dehalogenated product **21 a** in a lower yield than **NpMI** did (Figure 13 A). Bardagi and co‐workers recently reported conPET and e‐PRC reductions of 4‐bromobenzonitrile (*E* ^p^_red_=−1.95 V vs. SCE) using a modified naphthalene diimide precatalyst (**NDI‐c**).^[19m]^ The aryl(sp^2^) radical was trapped by an excess of benzene and afforded desired products (such as **21 b**) albeit in low yields (<20 %) (Figure 13 B). Though attention is needed to increase conversion and yields, this represents a potential alternative, milder set of conditions than transition‐metal‐free arylations of haloarenes requiring KO*t*Bu and organic additives. Such chemistry requires high temperatures to form electron donors in situ that initiate a base‐assisted homolytic aromatic substitution (BHAS) chain reaction.^[47]^

**Figure 14:**
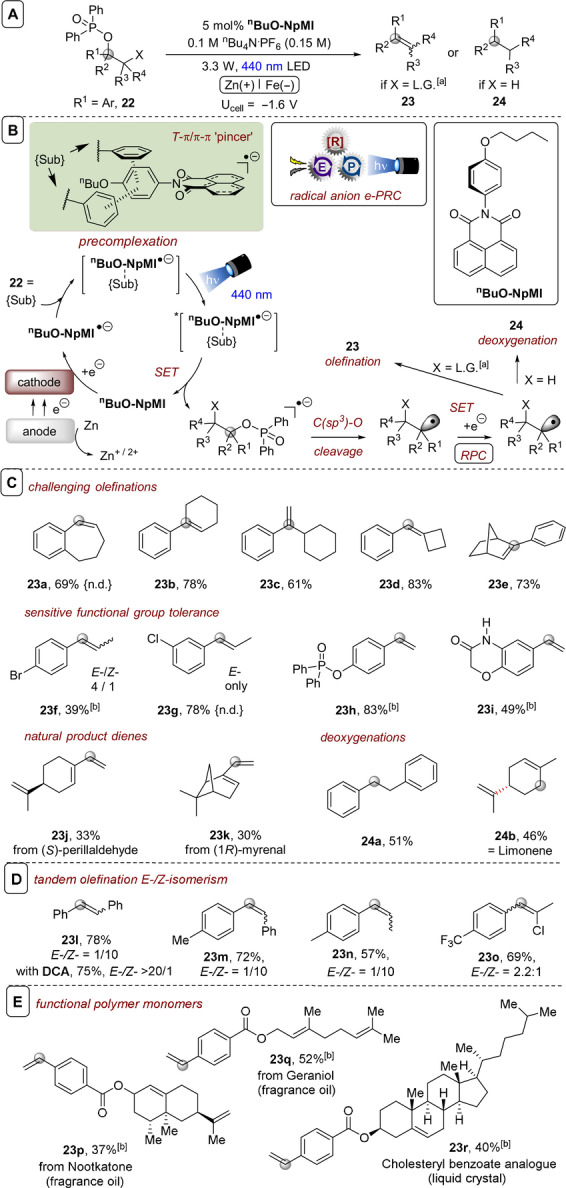
A) e‐PRC reduction of phosphinates of aliphatic alcohols with ^**n**^ **BuO‐NpMI**. B) Catalytic cycle including proposed precomplexation. C) Selected olefination examples. D) Tandem photoelectrochemical olefination *E*/*Z*‐isomerism. E) Application to functional polymer monomer synthesis. [a] Unless otherwise stated, phosphinate substrates with X=Cl. [b] From phosphinate with X=Br. n.d.: not detected.

**C(sp^3^)–O bond cleavages**: Inspired by previous photocatalytic generations of carbanions^[48]^ and direct electrolytic reductions of phosphinates in overall deoxygenations,^[49]^ Barham, König and co‐workers reported the first e‐PRC reductive cleavage of C(sp^3^)−O bonds to access sp^3^‐radicals and sp^3^‐centered carbanions.^[19q]^ Phosphinates of aliphatic alcohols successfully underwent e‐PRC reduction and C‐O cleavage when *N*‐(*para*‐butoxyphenyl)naphthalene monoimide (^**n**^ **BuO‐NpMI**) was employed (Figure 14 A). Following cathodic reduction to its radical anion and photoexcitation, *[^**n**^ **BuO‐NpMI^.−^**] engages phosphinates (*E* ^p^_red_ ≈−2.4 to −2.6 V vs. SCE) in SET. Then, C(sp^3^)−O bond cleavage of **22^.^** ^−^ forms a C(sp^3^) radical, proposed to undergo further SET reduction likely by cathodic current (or by *[^**n**^ **BuO‐NpMI^.^** ^−^]) to afford a C(sp^3^) carbanion. In the presence of an α‐leaving group (chloride, bromide), elimination occurs in an overall reductive olefination (Figure 14 B).

Since phosphinate substrates derive from α‐chloroketones and not aldehydes, the method has a different starting point to classic (Wittig‐type) olefinations that can be leveraged to access cyclic and hindered olefins (Figure 14 C). Compared to acid‐catalyzed or base‐dependent eliminations of alcohols, the method proceeds at ambient temperature, tolerates base‐sensitive functionality and allows user control over the site of olefination. For example, a substrate containing a free amide proton (**23 i**) was tolerated, as were esters (**23 p**–**r**). Formation of terminal olefin **23 c** demonstrates the benefit over an acid‐catalyzed elimination of a tertiary alcohol, which typically affords the most substituted olefin. In the absence of an α‐leaving group, overall deoxygenation occurred as a mild and tin‐free alternative to the Barton–McCombie reaction (Figure 14 B). The presence of a carbanion intermediate was confirmed when a phosphinate with a β‐chloro atom led to cyclopropanation.

Stilbene **23 l** could be accessed from the standard phosphinate **22**, but also from a cyclic phosphinate derived from a diol in a photoelectrochemical Corey–Winter‐type olefination reaction that avoids high temperatures and hazardous reagents normally associated with this reaction.

Unsymmetrical stilbenes could also be readily accessed by this method, surprisingly with generally high *Z*‐selectivities when ^**n**^ **BuO‐NpMI** was employed (Figure 14 D). Olefinations and deoxygenations of phosphinates derived from benzylic or allylic alcohols were successful, while those derived from non‐benzylic/allylic aliphatic alcohols did not react despite almost identical redox potentials. Remarkably, and in stark contrast to the previous report of Wickens,^[18c]^ aryl halides (chlorides and bromides) were tolerated under these conditions, despite their similar redox potentials to phosphinates (*E* ^p^_red_ (PhCl)= −2.78 V; *E* ^p^_red_ (PhBr)=−2.44 vs. SCE). A phenol‐derived phosphinate was also tolerated, contrasting with a previous reports on C(sp^2^)–O bond cleavage by PRC with a phenothiazine catalyst.^[50]^ 4‐Vinyl benzoates of terpene natural products that are liquid crystals or fragrance compounds could be prepared by late‐stage e‐PRC olefination from the phosphinates derived from the 4‐acetylbenzoate esters of the terpenes, giving rise to potential monomers for polymerization (Figure 14 E). Here, terminal olefination using base risks hydrolysis or E_2_ elimination, while direct esterification is problematic due to thermal instability of 4‐vinylbenzoic acid or its formulation with a radical stabilizer (BHT).

To probe the mechanism behind stilbene *E*/*Z*‐isomerism, control reactions with *E*‐stilbene as an input revealed the critical importance of e‐PRCat, light and potential on the *E*/*Z*‐isomerism. Luminescence spectroscopy revealed a nanosecond‐lived emitting state from [^**n**^ **BuO‐NpMI^.^** ^−^]* that was catalytically inactive in the initial SET reduction step (its lifetime was not quenched by **22 a**), but is likely responsible for *E*/*Z*‐isomerism. One candidate for this emitter is a quartet state, ^4^[^**n**^ **BuO‐NpMI^.^** ^−^]*, that results from intersystem crossing from a higher energy doublet state. The energy of this emitting state (*E* ^0‐0^) was identical to transition metal photocatalyst triplet energies known to effect *E*/*Z*‐photoisomerism of olefins by triplet–triplet energy transfer,^[51]^ and was within range of the triplet energies of stilbenes.

**Figure 15:**
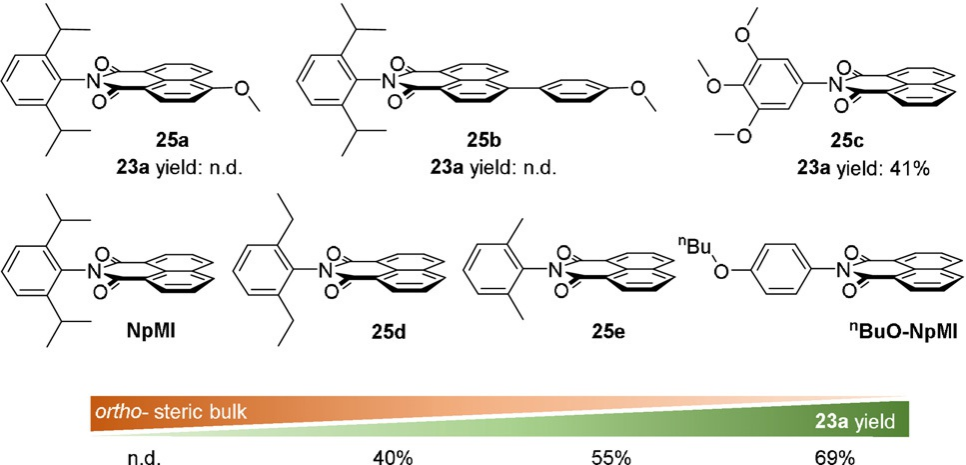
Relationship between catalyst structure and e‐PRC activity. n.d., not detected.

**Figure 16:**
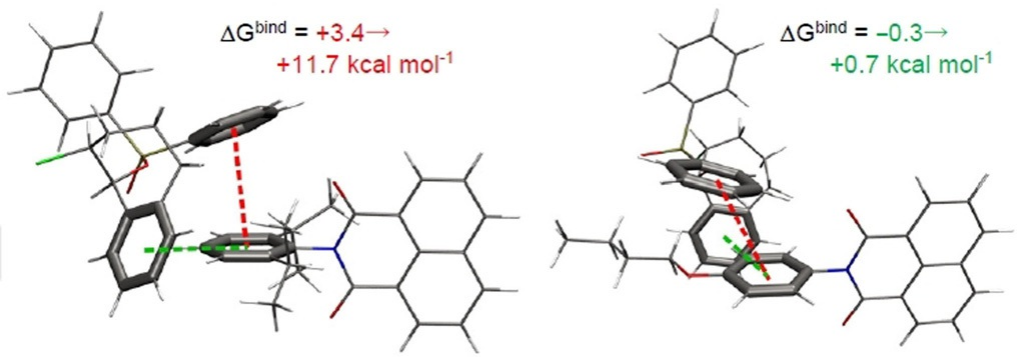
Examples of converged preassemblies for **22 a** with **NpMI^.^** ^−^ (left) or ^**n**^ **BuO‐NpMI^.^** ^−^ (right). Two candidates are shown, for others see Ref. [19q].

**Figure 17:**
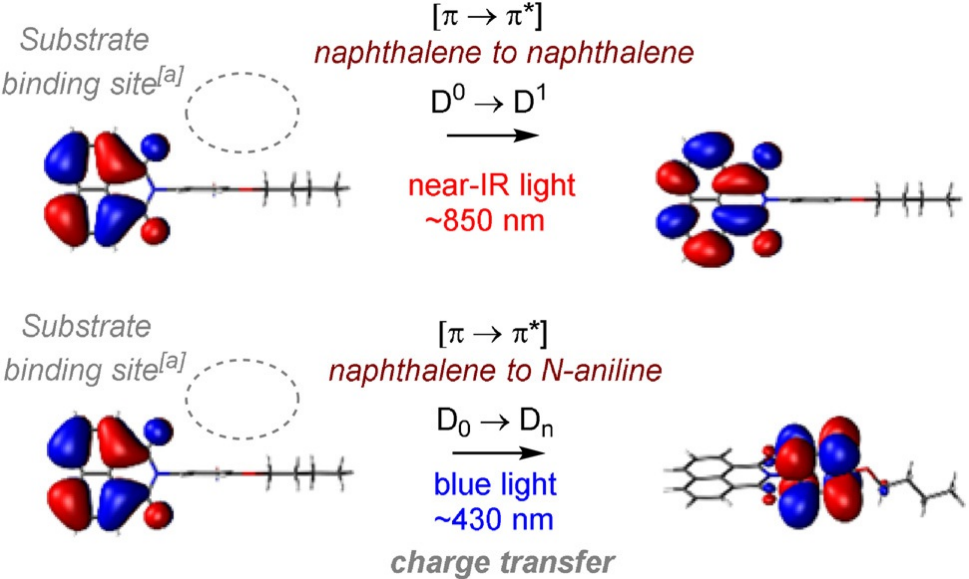
Orbital transitions for photoexcitation of ^**n**^ **BuO‐NpMI^.^** ^−^ corresponding to D_0_→D_1_/D_n_. [a] Binding site predicted by DFT calculations.

The authors sought to determine why ^**n**^ **BuO‐NpMI** was an effective catalyst for all (benzylic/allylic) substrates attempted, while **NpMI** was ineffective for most substrates, despite the identical ground‐state reduction potentials (*E*_1/2_=−1.3 V vs. SCE) and UV‐vis properties of both e‐PRCats, as their neutral or radical anion forms. By a combination of CV, EPR and computational investigations examining C(sp^3^)−O bond dissociation free energies (BDFEs), the authors found that the C(sp^3^)−O bond cleavage was likely the reactivity‐determining step, since the initial SET step was successful for both e‐PRCats. Mirroring their study on **TPA^.^** ^+^s,^[20]^ irradiation of the near‐IR UV‐vis bands of the catalyst gave no conversion, suggesting anti‐Kasha photochemistry from a higher‐order excited doublet state. Given 1) the known ultrashort lifetimes of similar excited‐state radical anions (*PDI^.^ ^−^, *NDI^.^ ^−^, Table 1) that prohibit their diffusion‐controlled photochemistry, 2) the fact that catalyst architecture was able to influence a C−O bond cleavage step and 3) the involvement of higher‐order excited doublet states, the authors proposed a preassembly of phosphinate substrates with ^**n**^ **BuO‐NpMI^.^** ^−^. However, in contrast to the authors’ earlier study on **TPA^.^** ^+^s,^[20]^ no spectroscopic (UV‐vis/EPR) changes were detected when electrogenerated ^**n**^ **BuO‐NpMI^.^** ^−^ was mixed with phosphinate **22 a**. The authors rationalized that precomplexation may occur at the *N*‐aryl moiety, which is not spectroscopically detectable since the spin density and chromophore of the radical anion are localized on the naphthalene moiety, orthogonal and electronically disconnected from the *N*‐aryl moiety. In support of this proposal, a strong correlation was found between decreasing steric hindrance at the *N*‐aryl moiety *ortho*‐positions of the e‐PRCats and increasing reactivity of **22 a** as a model substrate (Figure 15). Variations in the naphthalene moiety's electronics (e‐PRCats **25 a**,**b**) did not improve the activity compared to **NpMI**. An additional two alkoxy substituents at the *meta*‐positions (**25 c**) decreased activity relative to ^**n**^ **BuO‐NpMI**. DFT calculations (ωB97X‐D) found several candidate preassemblies between **22 a** and both **NpMI^.^** ^−^ and ^**n**^ **BuO‐NpMI^.^** ^−^. In all cases, converged structures resembled a “pincer” where two aromatic groups of **22 a** interact with the *N*‐aniline moiety by a *T*–π and a π–π interaction (Figure 16). Regardless of the converged candidate structure, binding free energies (Δ*G* ^bind^) were always more favourable for ^**n**^ **BuO‐NpMI^.^** ^−^ than for **NpMI^.^** ^−^. Finally, excited‐state calculations (DFT‐MCRI), in good agreement with experimental UV‐vis of ^**n**^ **BuO‐NpMI^.^** ^−^, revealed that the transition at ≈430 nm (D_0_→D_n_) involved a charge transfer from the naphthalene moiety to the *N*‐aniline moiety (Figure 17). Localization of electron density at the *N*‐aniline in this excited state (D_n_) is thus exactly where required for rapid intra‐assembly SET. A more intimate precomplexation of **22 a** and ^**n**^ **BuO‐NpMI^.^** ^−^ likely promotes C(sp^3^)–O bond cleavage.

**Figure 18:**
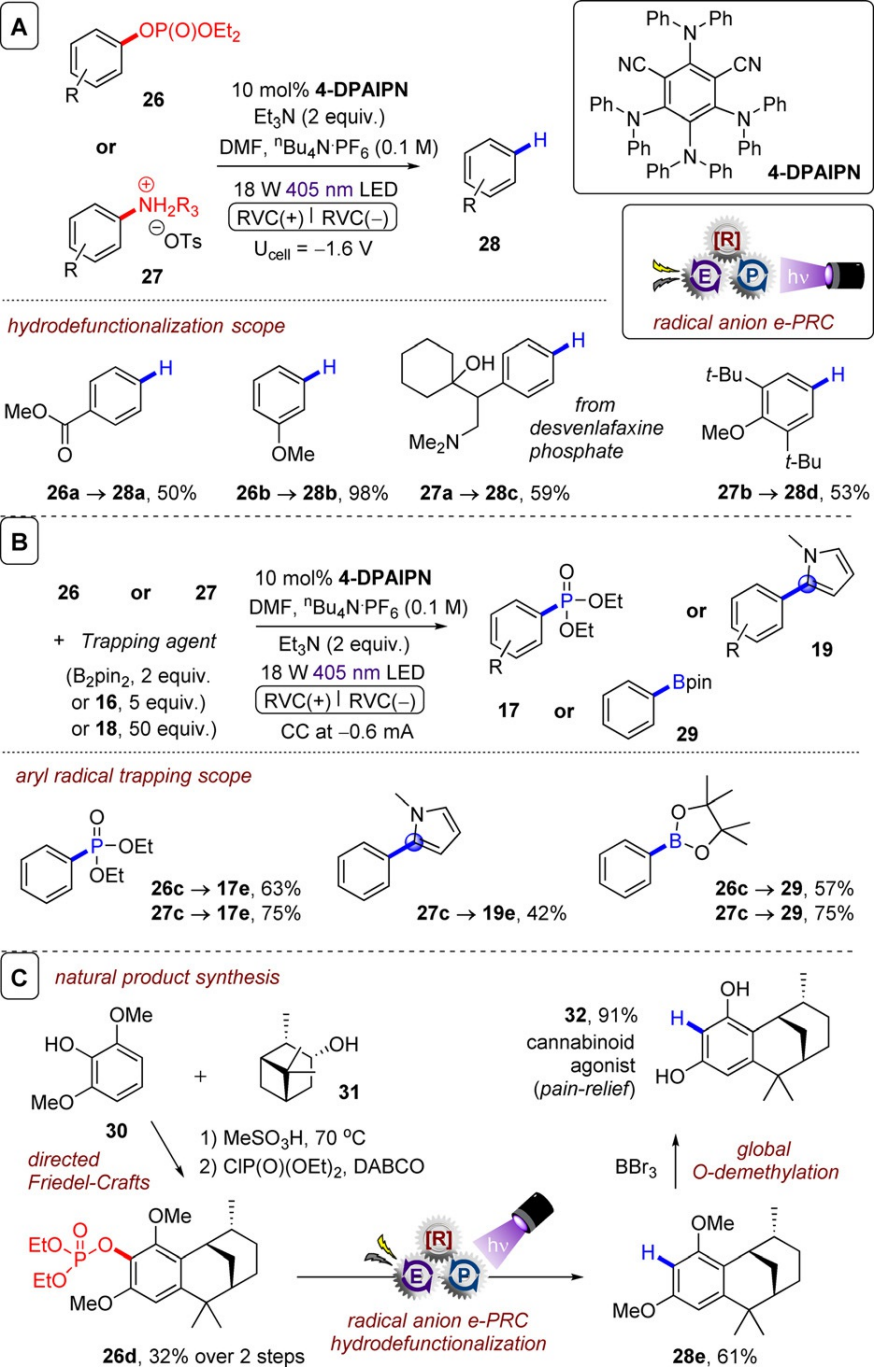
e‐PRC reduction of phosphonated phenols and anilinium salts. A) Substrate scope of hydro‐defunctionalization. B) Substrate scope of aryl radical trapping. C) Application in alkali metal‐free synthesis of a cannabinoid agonist.

**Scheme 2:**
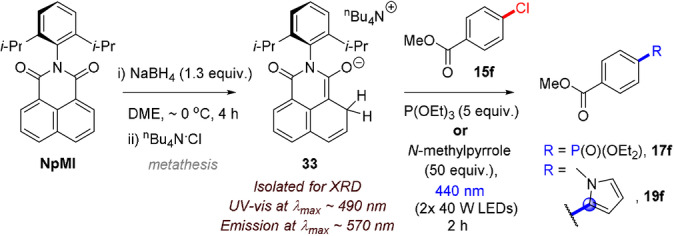
Chemical reduction of **NpMI** affords a hydride adduct which serves as a stoichiometric photoreductant in the coupling of an activated aryl chloride.

**C(sp^2^)–O and C(sp^3^)–^+^NR_3_ bond cleavages**: Wickens and co‐workers recently extended their aryl halide reduction methodology to the reduction of aryl pseudohalides.^[19t]^ Phosphonated phenols and anilinium salts could be reduced by e‐PRC in an overall hydro‐defunctionalization reaction as the main theme of the study (Figure 18 A). Alternatively, the aryl radical could again be intercepted by triethylphosphite, *N*‐methylpyrrole or B_2_pin_2_ (Figure 18 B), inspired by the authors’ previous work^[18c]^ and that of the groups of Larionov^[50]^ and König,^[52]^ who demonstrated reductive cleavages of strong bonds and borylation of aryl radicals via photocatalysis involving proton‐coupled electron transfer^[50]^ or an EDA complex.^[52]^ Reactions were conducted in divided H‐cells under a constant potential, with 10 mol % e‐PRCat and 2 equiv. of Et_3_N present in the cathodic chamber as a terminal reductant. As an example synthetic application, a phenol was used to direct the Friedel–Crafts reaction of **30** with **31** followed by e‐PRC hydro‐defunctionalization to **28 e** and global O‐demethylation to afford tricyclic resorcinol cannabinoid agonist **32** in 14 % yield over three steps. This application followed the work of Makriyannis and co‐workers^[53]^ but substituted dissolving Li metal reduction with e‐PRC. Although isophthalonitrile structures are widely employed in PRC, this report,^[19t]^ together with a concurrently reported conPET variant,^[19s]^ constitute the first reports of isophthalonitrile radical anions in reductive catalytic transformations. **4‐DPAIPN** structurally resembles cyano‐substituted triarylamines used in oxidative e‐PRC;^[20]^ it is interesting to find the acyclic triarylamine architectural family can be used in both reductive and oxidative e‐PRC reactions. Current‐based analysis of the reaction rate over time evidenced the enhanced stability of **4‐DPAIPN** compared to previously reported catalyst (**NpMI**). **NpMI** decomposed over time under the reaction conditions, decreasing conversion rate. As the reaction progressed, the rate increased again, meaning a decomposed form of the catalyst is also catalytically (albeit less) active. This concurs with Nocera and co‐workers’ recent report^[19r]^ which analysed the electro‐decomposition product of **NpMI** (irreversible CV wave at −2.3 V). Electrolysis at U_cell_=−3.0 V provided a species absorbing at (*λ*_max_=)480–500 nm and luminescing at (*λ*_max_=)560–580 nm, which matched the spectra of a species formed when **NpMI** was chemically treated with NaBH_4_ (in DME) or TBAF (in DMAc). XRD of the species chemically formed with NaBH_4_ revealed a hydride adduct **33** (Scheme 2). Irradiating **33** with 440 nm in the presence of stoichiometric 4‐methylchlorobenzoate (**15 f**) and excesses of P(OEt)_3_ or *N*‐methylpyrrole led to detection of **17 f** and **19 f** by ^1^H NMR spectroscopy, confirming that **33** can serve as a photoreductant. Nocera questioned the previously proposed involvement of radical anion photocatalysts,^[18c]^ claiming that **NpMI^.^** ^−^ is too short‐lived (*τ*=24 ps) to permit diffusion‐controlled photochemistry, yet the electro‐decomposed emitting species assigned as **33** is sufficiently long‐lived to do so (*τ*=20 ns). However, photoreductive activity of **NpMI^.^** ^−^ is not ruled out since Wickens’ kinetic analysis clearly evidenced the presence of multiple (at least two) active catalysts during e‐PRC reactions.^[19t]^ Although the excited state of **33** was effectively quenched by activated aryl chloride **15 f**, PhCl was ineffective (radical trapping of PhCl was not reported). Adduct **33** or related species may be a candidate for the emitting state (ES_1_) in Barham, König and co‐workers’ study,^[19q]^ but comparisons are still questionable due to 1) UV‐vis absorptions (*λ*_max_) of **NpMI^.^** ^−^ agreeing within ±10 nm despite different solvents (DMAc^[19r]^ vs. MeCN) in the two studies,^[19q]^ yet ES_1_ differing in peak shape and *λ*_max_ (by ca. 40 nm);^[19q]^ 2) notably lower cell potentials for electrolysis (U_cell_=−1.6 V) used both in spectroscopy and reactions where U_cell_=−3.0 V led to intractable complex mixtures.^[19q]^ The proposal of diffusion‐controlled SET photochemistry of adducts like **33**,^[19r]^ while intriguing, cannot rationalize 1) clear structure/activity relationships at the *N*‐aniline of **NpMI**‐type catalysts,^[19q]^ 2) reduction of non‐activated aryl chlorides like PhCl/4‐chloroanisole (**15 a**),^[18c]^ 3) clear quenching of PRCat**^.^** ^−^s UV‐vis/EPR signals upon irradiation in the presence of substrates[^4a^, ^19q^, ^19s^] and 4) absence of quenching of longer (ns or μs)‐lived emitting species derived from radical anion PRCat**^.^** ^‐^s in other reports.[^4g^, ^19q^] Closed‐shell anionic species may be reservoirs/precursors to radical anion photocatalysts as proposed by Miyake and co‐workers.^[4g]^

**Future perspectives**: Radical ion e‐PRC is a rapidly expanding field that offers 1) new opportunities in synthetic transformations, including complex molecule functionalizations, 2) access to ultrahigh‐energy redox processes under exceedingly mild conditions to cleave or form strong bonds, and 3) new opportunities for selectivity that differ from conventional parameters in SOE and PRC (redox potentials). While radical ion catalysts and their excited‐state behaviours are likely interchangeable concepts between conPET and e‐PRC manifolds, downstream chemistry of a target substrate following its initial SET differs. Here, e‐PRC offers the attractive and unique benefit of user‐potentiocontrollable radical polar crossover. With the key importance of preassembly in radical ion photocatalysis established, a particularly exciting prospect is leveraging factors in preassembly formation to guide 1) SET chemoselectivity and/or 2) following bond cleavages/formations; similar to the “lock‐and‐key” concept of enzyme catalysis. Indeed, noncovalent interactions (dispersion, π–π stacking) historically viewed as “weak interactions” are receiving increasing attention in catalysis.^[54]^

Another emerging theme is access to excited states higher than the first excited state in anti‐Kasha photochemistry.[^4g^, ^19q^, ^20^] This phenomenon in itself corroborates substrate/photocatalyst preassembly to rationalize SET taking place more rapidly than internal conversion of the higher‐order excited state (D_n_→D_1_). Thereby, anti‐Kasha radical ion photochemistry harnesses the full visible photon redox energy, where in conventional PRC much is lost to internal conversion. Consequentially, the redox window of transformations is dramatically expanded—substrates beyond typical solvent windows (MeCN/DMF) are engaged. The ultrafast timescale within which SET must occur shields the bulk reaction mixture from extreme redox potentials generated in situ—a clear benefit compared to high cell potentials constantly applied across reaction mixtures in direct electrolysis.

In theory, preassembly and anti‐Kasha photochemistry should raise photochemical reaction (quantum) efficiency. In practice, long reaction times often plague radical ion e‐PRC reactions. A greater understanding of factors involved in the preassembly is essential for the field to achieve higher quantum efficiencies. While it is straightforward to assign quantum yields and lifetimes to closed‐shell excited states, interrogation of open‐shell doubled excited states is a challenging endeavour demanding more sophisticated spectroscopic and theoretical techniques. This said, solvated electrons^[4g]^ and decomposition of radical ion PRCats to closed‐shell PRCats as in *DCA^.^ ^−^ and *NpMI^.^ ^−[19p,r]^ must be probed as alternative mechanisms as the field continues to evolve. Finally, limited reports of scalability in radical ion e‐PRC are likely due to practitioners requiring 1) divided cell configurations to spatially mitigate nonproductive half‐reactions and 2) constant potential desirable for selective e‐PRCat activation.

## 2 Photocatalyst Electro‐recycling

**Figure 19:**
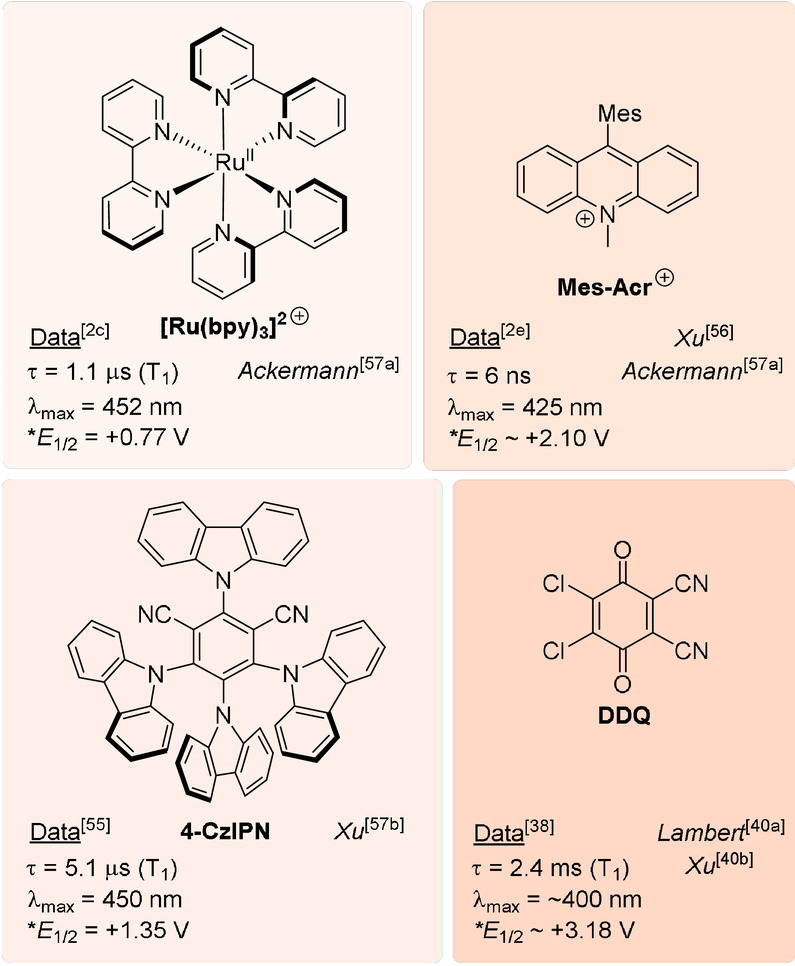
PRCats reported in recycling e‐PRC. Red shading is indicative of oxidizing power. Selected photophysical and redox potential data are shown.

**Figure 20:**
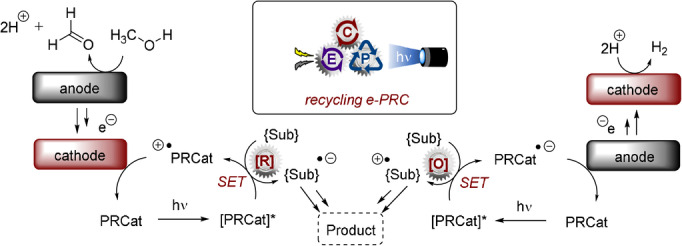
Concept of photocatalyst electro‐recycling (recycling e‐PRC).

The second subcategory of e‐PRC, dubbed “recycling e‐PRC”, involves the turnover of a photocatalyst that is a known photoredox catalyst (PRCat) in PRC, and that is a colored species in its ground, neutral state. Figure 19 depicts the structures of PRCats used in recycling e‐PRC; their photophysical properties are thoroughly detailed elsewhere.[^2c^, ^2e^, ^38^, ^55^] In recycling e‐PRC, the available “redox window” is no wider than it is for PEC. Instead, the key benefit is the replacement of sacrificial oxidants or reductants in the photocatalyst turnover with electrochemistry (Figure 20), which can (or whose by‐products can) interfere with downstream chemistry and/or complicate separation of desired products.[^9^, ^56^, ^57^] This is not to say that sacrificial oxidants or reductants are completely avoided; they can be required by the counter‐electrode's reaction. However, here protons or water typically serve as much milder, atom‐economical sacrificial oxidants or reductants (respectively). Moreover, a divided cell configuration provides the opportunity to spatially separate sacrificial redox agents from the desired catalytic reaction in the product‐forming chamber.

In a seminal report that was previously reviewed elsewhere,[^9^, ^10^] Xu and co‐workers reported recycling e‐PRC using Fukuzumi's catalyst **Mes‐Acr^+^** for a Minisci‐type coupling of alkyltrifluoroborates with heteroarenes in an undivided cell.^[56]^ Inspired by this report, different applications of recycling e‐PRC have emerged in recent years.[^40^, ^57^] Recycling e‐PRC benefits from the use of well‐characterized and long‐lived (nanosecond to millisecond) closed‐shell excited states. As opposed to radical ion e‐PRC where divided cells are generally employed, recycling e‐PRC typically uses undivided cells or modular batch/recirculated flow setups. In fact, most reports in recycling e‐PRC easily achieve gram‐ to multigram‐scale reactions—indicating the practical accessibility and robustness of this synthetic photoelectrochemical technology.

**Figure 21:**
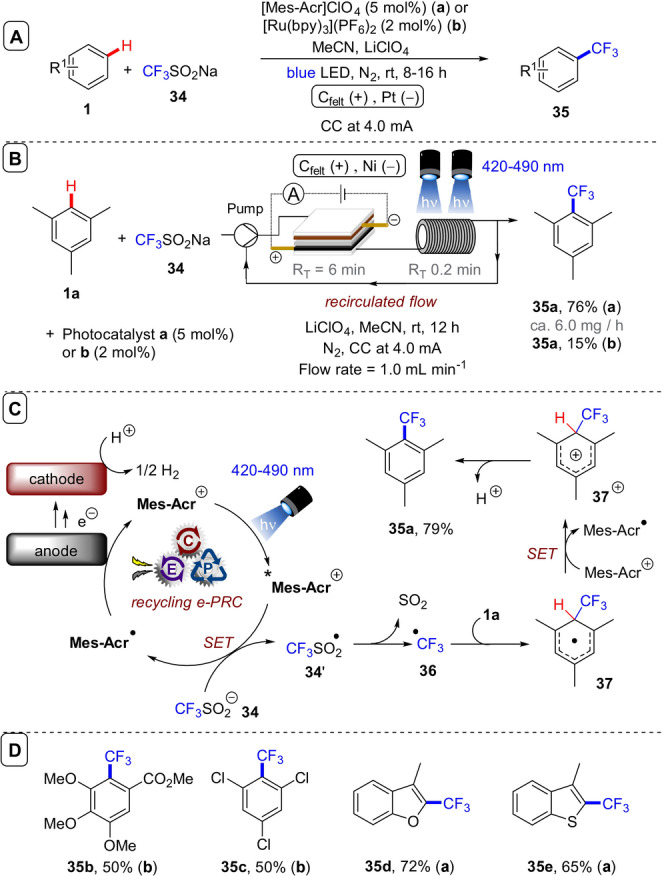
A) C(sp^2^)–H trifluoromethylation using CF_3_SO_2_Na, graphite felt (C_felt_) anode (Pt cathode) and a photocatalyst. B) C−H trifluoromethylation in a flow setup with flow rate 1.0 mL min^−1^ (*R*_T_ “per pass”=6 min). C) Proposed mechanism. D) Selected substrate scope.

**C(sp^2^)–H trifluoromethylation**: In an elegant example of photocatalyst electro‐recycling, Ackermann and co‐workers reported the PEC C(sp^2^)–H trifluoromethylation of arenes and heteroarenes under anodic current and with Langlois’ reagent, CF_3_SO_2_Na (**34**) (Figure 21).^[57a]^ Upon visible‐light photoexcitation of the Fukuzumi‐type organic dye, **Mes‐Acr^+^** (catalyst **a**) the authors proposed that the excited‐state ^*^ **Mes‐Acr^+^** engages in SET with the CF_3_SO_2_ anion **34** to furnish the reduced acridinyl radical form of the catalyst and the CF_3_SO_2_ radical **34**′. Loss of SO_2_ generates the active trifluoromethyl radical **36**, which attacks arene substrate **1 a** to form arene radical **37**. SET oxidation of **37**, either by **Mes‐Acr^+^** or by the anode, forms cation **37** ^+^ which loses a proton to form the trifluoromethylated product **35 a**. The protons generated could undergo cathodic reduction to form H_2_ to complete the circuit. Meanwhile, ground‐state **Mes‐Acr^+^** photocatalyst is then regenerated by anodic oxidation of its acridinyl radical form at the C_(felt)_ anode under constant current conditions (4.0 mA). LiClO_4_ was selected as an electrolyte which eases the separation of products, while another set of conditions were developed using **Ru(bpy)_3_** ^**2+**^ (catalyst **b**). This method enabled the C(sp^2^)–H trifluoromethylation of a range of arenes including electron‐rich and electron‐poor arenes and various heteroarenes (affording products such as **35 b**–**35 e**, Figure 21 D). As a pioneering example of the transfer of PEC to a continuous flow setup, an electrochemical flow coil consisting of a carbon felt (C_felt_) anode and a nickel cathode was employed prior to a photochemical fluoropolymer coil added in sequence (Figure 21 B). Recirculation of the reaction mixture over 12 h afforded **35 a** in 76 % yield using catalyst **a** (conditions employing catalyst **b** were inferior). Speaking to the facile integration of continuous flow with process analytical technologies (PATs),^[58]^ the authors used an in‐line NMR spectrometer to monitor the reaction and observed the Wheland‐type arenium cation **37** ^+^ as a long‐lived intermediate via ^19^F and ^1^H NMR spectroscopy. This demonstrated a key opportunity for continuous flow in the investigation of SET reaction mechanisms by its ability to provide a steady‐state concentration of reactive species.

**Figure 22:**
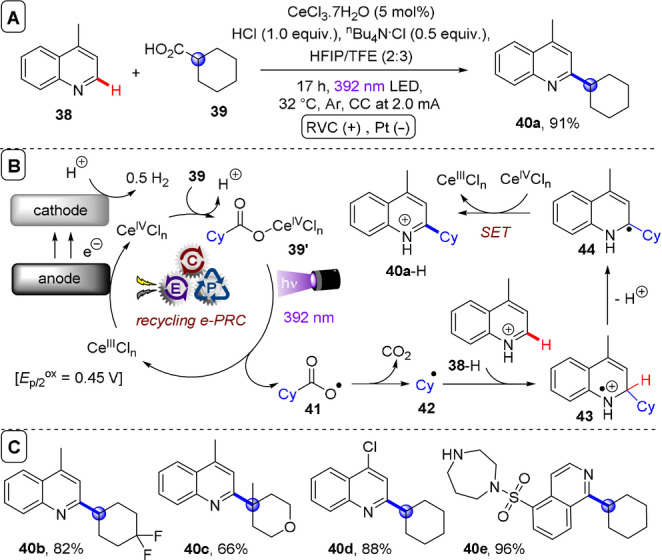
A) Decarboxylative C−H alkylation of heteroarenes using RVC anode (Pt cathode) and CeCl_3_⋅7 H_2_O. B) Proposed mechanism. C) Selected scope.

**Figure 23:**
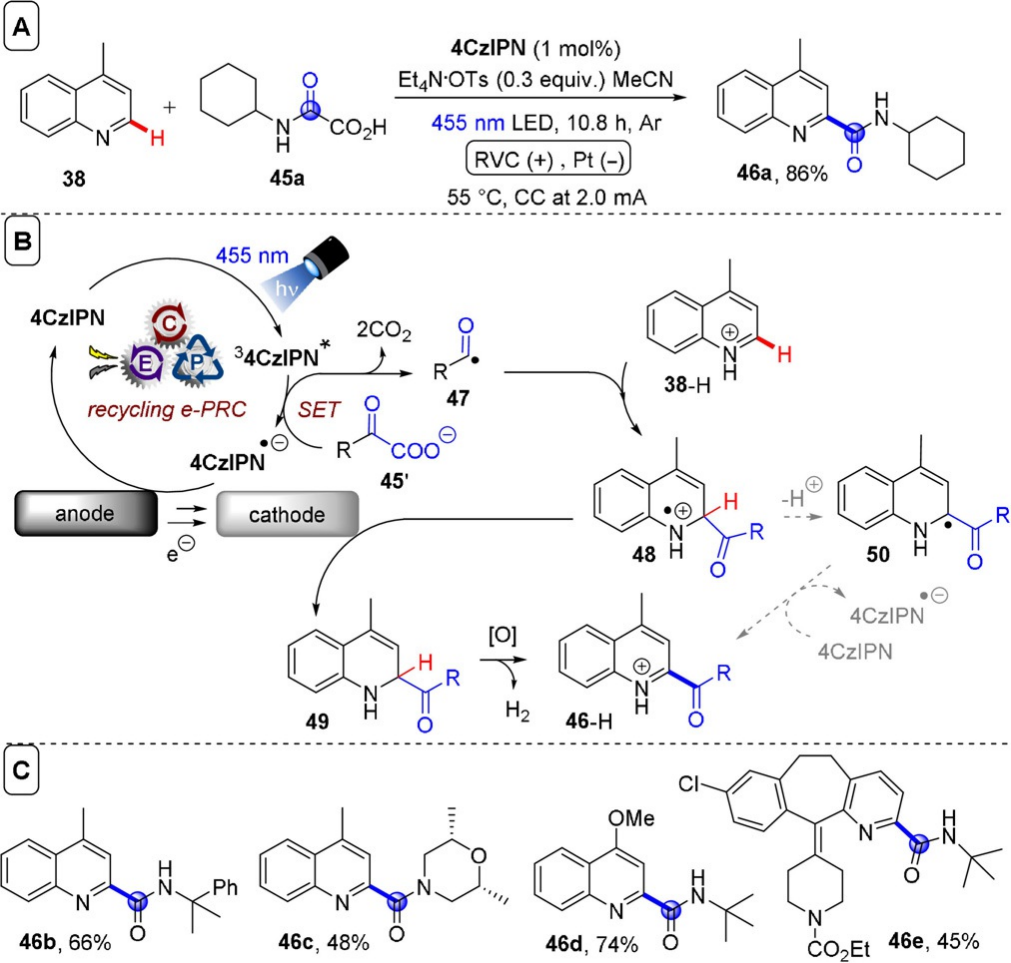
A) Decarboxylative C−H carbamoylation of heteroarenes using RVC anode (Pt cathode) and **4CzIPN** as photocatalyst. B) Proposed mechanism. C) Selected scope.

**Figure 24:**
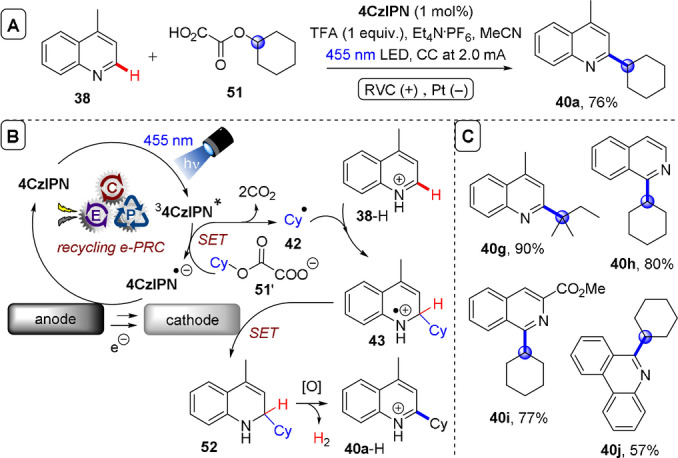
A) C−H alkylation of heteroarenes with alkyl oxalates using RVC anode (Pt cathode) and **4CzIPN** as photocatalyst. B) Proposed mechanism. C) Selected scope.

**C(sp^2^)–C(sp^3^) coupling**: Building upon their previous work in photoelectrochemical C(sp^2^)–C(sp^3^) Minisci‐type coupling of heteroarenes **30** with alkyl trifluoroborates,^[56]^ and as the first synergy of PEC with cerium photocatalysis,^[59]^ Xu and co‐workers reported direct decarboxylative C−H alkylation of heteroarenes **38** using an RVC anode and CeCl_3_⋅7H_2_O as a photocatalyst precursor (Figure 22).^[57b]^ Initial anodic oxidation of Ce^III^ to Ce^IV^ occurs (Figure 22 B), followed by coordination of Ce^IV^ by the carboxylic acid **39** to form complex **39′**. Photoinduced ligand‐to‐metal charge transfer (LMCT) regenerates Ce^III^ and simultaneously forms carboxyl radical **41**, which decarboxylates to afford alkyl radical **42**. Given the protic reaction conditions, the authors proposed the addition of alkyl radical **42** to protonated substrate **38**‐H to give transient radical cation **43**, which then loses a proton to form **44**. A highly exothermic oxidation of **44** by Ce^IV^ then affords product **40 a**‐H. The substrate scope demonstrated that various examples of carboxylic acids (primary, secondary, tertiary and α‐alkoxy and aliphatic) could be tolerated, and the power of the method was demonstrated by the late‐stage functionalizations of various *N*‐heteroarenes including bioactive molecules such as **40 e** (fasudil), a Rho‐associate protein kinase inhibitor (Figure 22 C).^[60]^ In the same report, Xu and co‐workers further exploited decarboxylative radical formation in the PEC carbamoylation of heteroarenes using an RVC anode and a **4CzIPN** photocatalyst (Figure 23).^[57b]^ Upon photoexcitation of **4CzIPN** with blue LEDs, SET oxidation of oxamate substrate **45**′ by ^3^ 4CzIPN* forms **4CzIPN^.−^** and, upon decarboxylation, carbamoyl radical **47** which adds to protonated substrate **38**‐H resulting in radical cation **48** (Figure 23 B). Reduction of **48**, either by the Pt cathode or by **4CzIPN^.^** ^−^, affords intermediate **49**. Anodic oxidation of **49** with loss of H_2_ was proposed to result in aromatization to protonated product **46**‐H. Alternatively, the authors proposed deprotonation of **48** would afford radical **50** which could be oxidized by ground‐state **4CzIPN** or by the anode. The substrate scope featured various examples of oxamic acids (bearing primary, secondary and tertiary *N*‐substituents) and various electron‐deficient *N*‐heteroarenes (affording products such as **46 a**–**e**), including the late‐stage functionalization of antihistamine Loratadine^[61]^ to afford **46 e** (Figure 23 C). Finally, Xu and co‐workers extended their method to alkyl oxalates as precursors to alkyl radicals in the absence of a transition metal catalyst (Figure 24).^[57c]^ Photoexcited **4CzIPN** oxidizes alkyl oxalate **51** to generate alkyl radical **42** via double decarboxylation. The authors proposed similar downstream chemistry (Figure 24 B) to that in the case of the carbamoyl radical (Figure 22) to afford **40 a‐H**. Although the reaction was amenable to various examples of secondary and tertiary oxalates (Figure 24 C), primary oxalates were ineffective as alkyl radical precursors.

**Figure 25:**
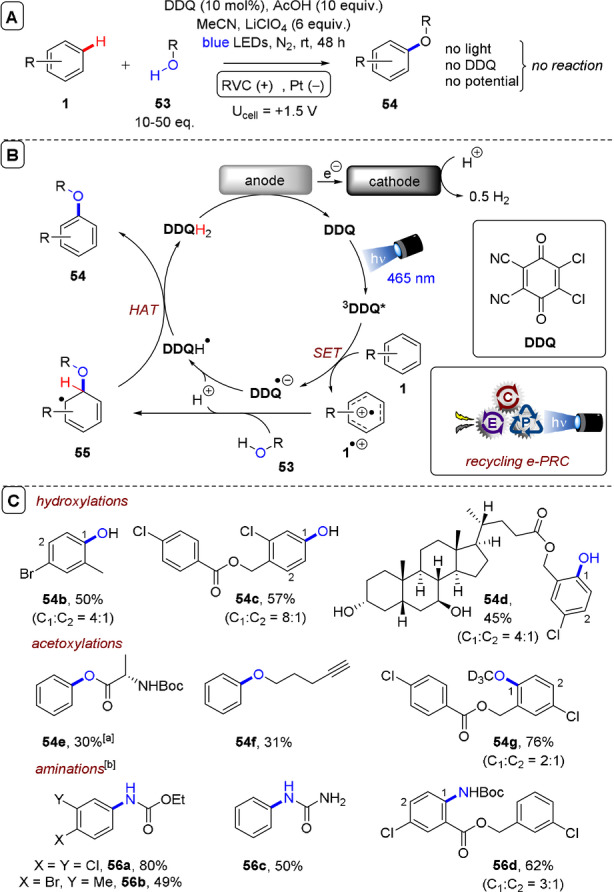
A) C(sp^2^)–H acetoxylation of arenes a using an RVC anode (Pt cathode) and **DDQ** as photocatalyst. B) Proposed mechanism. C) Selected substrate scope. [a] Boc‐Ala‐OH substituted for the AcOH additive. [b] 3–15 equiv. of amide/carbamate nucleophile was used.

**C(sp^2^)–O/N bond formations**: So far, examples covered have focused on C(sp^2^)−C bond formations. However, C(sp^2^)−O and C(sp^2^)−N bond formations can also be achieved under recycling e‐PRC. **DDQ** as a neutral photocatalyst is a powerful oxidant in its long‐lived triplet excited state (+3.18 V vs. SCE).^[38]^ Despite its absorbance maxima at ≈400 nm, **DDQ** is successfully photoexcited with longer wavelength blue (455 nm) light. Prior to any contemporary reports of recycling e‐PRC, König's group achieved the photocatalytic oxidation of electron‐deficient arenes by ^3^ DDQ* in the presence of *tert*‐butyl nitrite and molecular oxygen,^[39]^ which was reviewed previously.^[9]^ Inspired by this work, and by their earlier work on recycling e‐PRC for the S_N_Ar‐type arene azolations^[62]^ (reviewed previously),^[9]^ the Lambert group recently reported arene acetoxylation using **DDQ** as photocatalyst under recycling e‐PRC (Figure 25).^[40a]^ Benzene (**1 e**) was hydroxylated to phenol (**54 a**) in the optimization study. Control reactions confirmed the necessity of light, catalyst and potential in the reaction (Figure 25 A). Direct electrolysis at a potential of U_cell_=+3.2 V gave no product after 48 h (presumably, decomposition occurred under direct electrolysis, as previously reported in the radical ion e‐PRC azolation of benzene),^[18a]^ confirming the superiority of photoelectrochemistry in this reaction.

**Figure 26:**
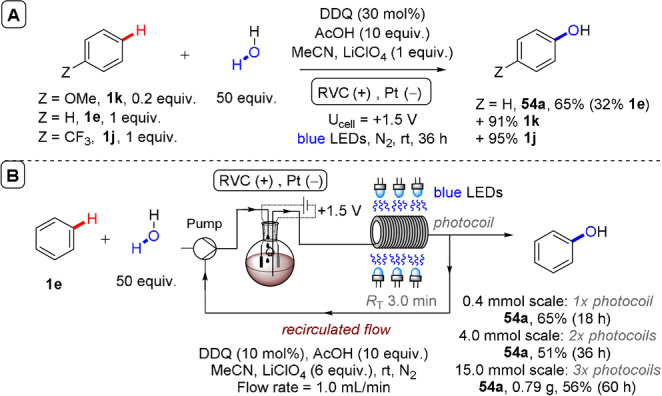
A) Competition experiments between arenes with different electronics in recycling e‐PRC hydroxylation. B) Recirculated flow recycling e‐PRC hydroxylation of benzene.

In the mechanism, SET oxidation of arene **1** by ^3^ DDQ* was proposed (Figure 25 B), followed by nucleophilic addition of heteroatom partner **53** to radical cation **1^.^** ^+^. **DDQ^.^** ^−^ formed after SET is protonated and engages **55** in HAT to afford product **54**. A range of electron‐deficient arenes were hydroxylated and acetoxylated to afford products **54 a**–**g** in modest to very good (30–76 %) yields (Figure 25 C). Remarkable selectivities were observed: aliphatic alcohols, terminal alkynes and benzylic positions were all tolerated, where these positions would not likely tolerate direct electrolysis. Aminations were also possible with free amides, carbamates and ureas, affording products **56 a**–**d** in satisfactory to high (49–80 %) yields, although the scope of amination partner was relatively narrow in each of these classes. In an intriguing competition experiment (Figure 26 A), benzene (**1 e**) was selectively hydroxylated while anisole (**1 k**) and trifluorotoluene (**1 j**) were untouched by the recycling e‐PRC conditions. This highlights the importance of matching excited‐state PRCat redox potentials to the substrate. Trifluorotoluene is beyond the scope of ^3^ DDQ* (and *TAC^.2^ ^+^, >3.3 V vs. SCE, but could be engaged by potent *TdCBPA^.^ ^+^, up to +4.4 V vs. SCE). On the other hand, anisole is easily accessible and likely oxidized by ^3^ DDQ*. It is plausible that back electron transfer (BET) is rapid enough to prohibit downstream chemistry of **1 k^.^** ^+^.^[63]^

In general, this recycling e‐PRC was superior to the preceding PRC‐only report^[39]^ where cheap constant potential obviated the need for the sacrificial oxidant (molecular oxygen) and expensive *tert*‐butyl nitrite, both hazardous reactants that require considerable safety considerations for scaling up.^[64]^ In this respect, the Lambert group successfully scaled the benzene‐to‐phenol hydroxylation reaction in a recirculated continuous flow setup (Figure 26 B). Electro‐regeneration of **DDQ** was achieved in a batch undivided cell, while the reaction mixture was recirculated through blue LED‐irradiated coil with a residence time (*R*_T_) of 3 min “per pass”. By extending reaction time and adding additional photocoils, the reaction was successfully scaled from 0.4 to 15 mmol without appreciable loss in the yield of **54 a**. Thereby, recycling e‐PRC benefitted safety and cost‐efficiency.

**Figure 27:**
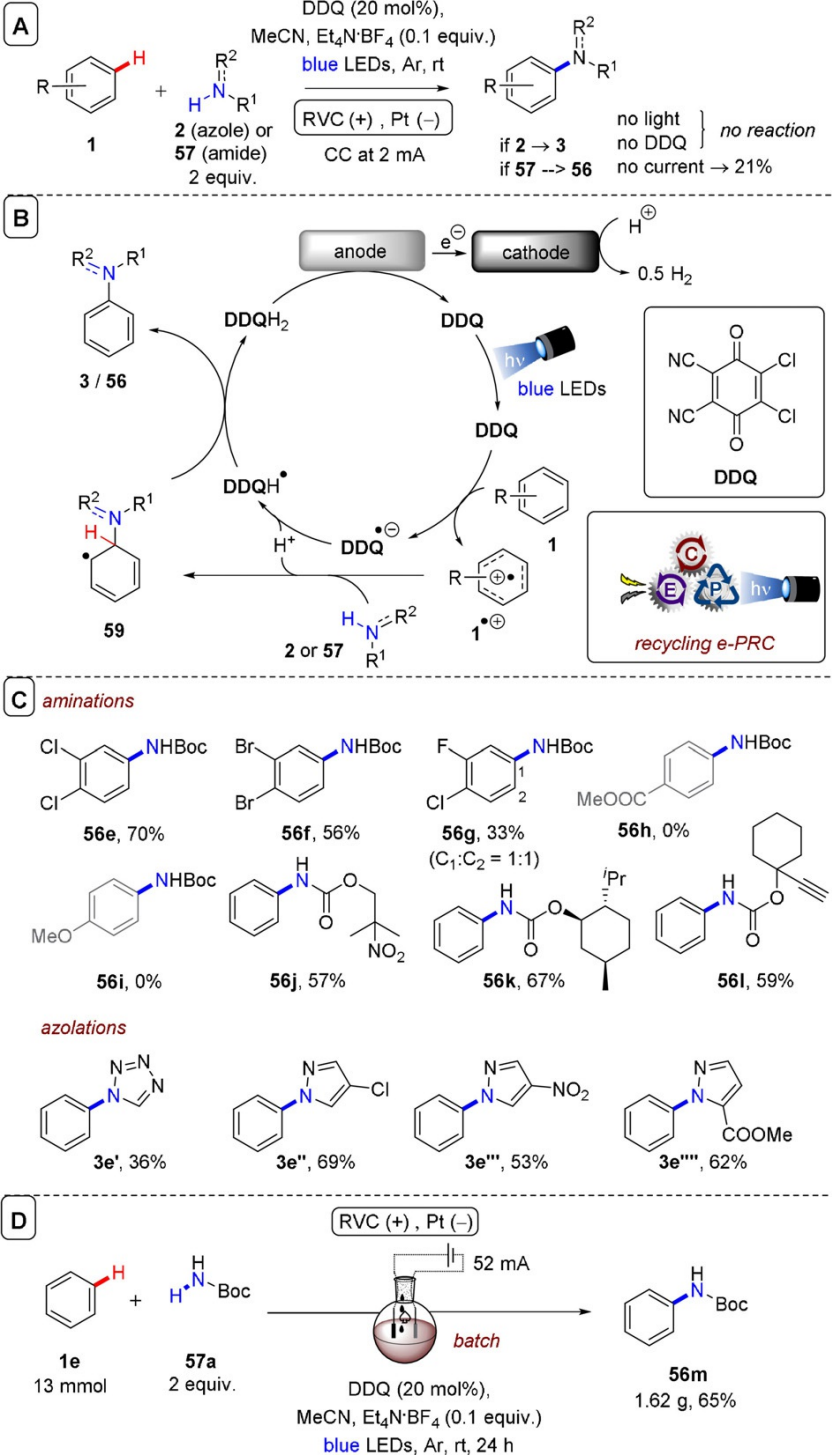
A) C(sp^2^)–H amination of arenes with carbamates using an RVC anode (Pt cathode) and **DDQ** as photocatalyst. B) Proposed mechanism. C) Selected substrate scope. D) Gram‐scale batch reaction.

Xu and co‐workers also reported arene heteroamination using **DDQ** under recycling e‐PRC conditions (Figure 27 A).^[40b]^ Compared to the Lambert group's report, although conditions employed a higher loading of **DDQ** (20 mol %), loadings of electrolyte (0.1 equiv.) and amination partner (2 equiv.) were markedly lower, possibly due to the use of constant current (2 mA) to drive the reaction. The focus was on aminations; hydroxylations and acetoxylations were not investigated. The mechanism was as aforementioned (Figure 27 B). A much broader scope of amination partners was reported, including azolations, affording products in modest to high (36–70 %) yields (Figure 27 C). A gram‐scale batch reaction worked (Figure 27 D). Like Lambert's report,^[40a]^ anisole was unreactive. ^3^ DDQ* did not engage methyl benzoate—the upper redox limit was dihalobenzenes.

Although these conditions^[40b]^ were unable to engage very electron‐deficient arenes (radical ion e‐PRC with **TPA**s were able to engage up to acetophenone),^[20]^ the yields of azolated dihaloarenes were higher than in radical ion e‐PRC reports[^18a^, ^20^] and, notably, arene **1** was the limiting reagent as opposed to the requirement of excess arene in previous oxidative radical ion e‐PRC (typically 1 mL)[^18a^, ^20^] and conPET (typically 8 mL)^[4]^ reports. The excess arene required in radical cation e‐PRC is likely due to the requirement of precomplexation between ground‐state radical cation and arene for successful photochemistry, whereas ^3^ DDQ* is long‐lived enough to engage in outer‐sphere diffusion‐controlled photochemistry. By increasing the electrode surface area and applying a higher constant current (52 mA), Xu and co‐workers scaled the reaction up to produce gram quantities of **56 m** deriving from the amination of benzene (Figure 27 D) without any flow setup. To shed light on the regioselectivity of nucleophilic addition, Xu and co‐workers performed DFT calculations on the radical cations of haloarenes. Calculated LUMOs showed, in all cases, that positions *para* to the halogens had larger orbital coefficients than other positions, rationalizing for the first time regioselectivity for the nucleophilic addition in these radical cation S_N_Ar reactions of electron‐deficient arenes.

**Figure 28:**
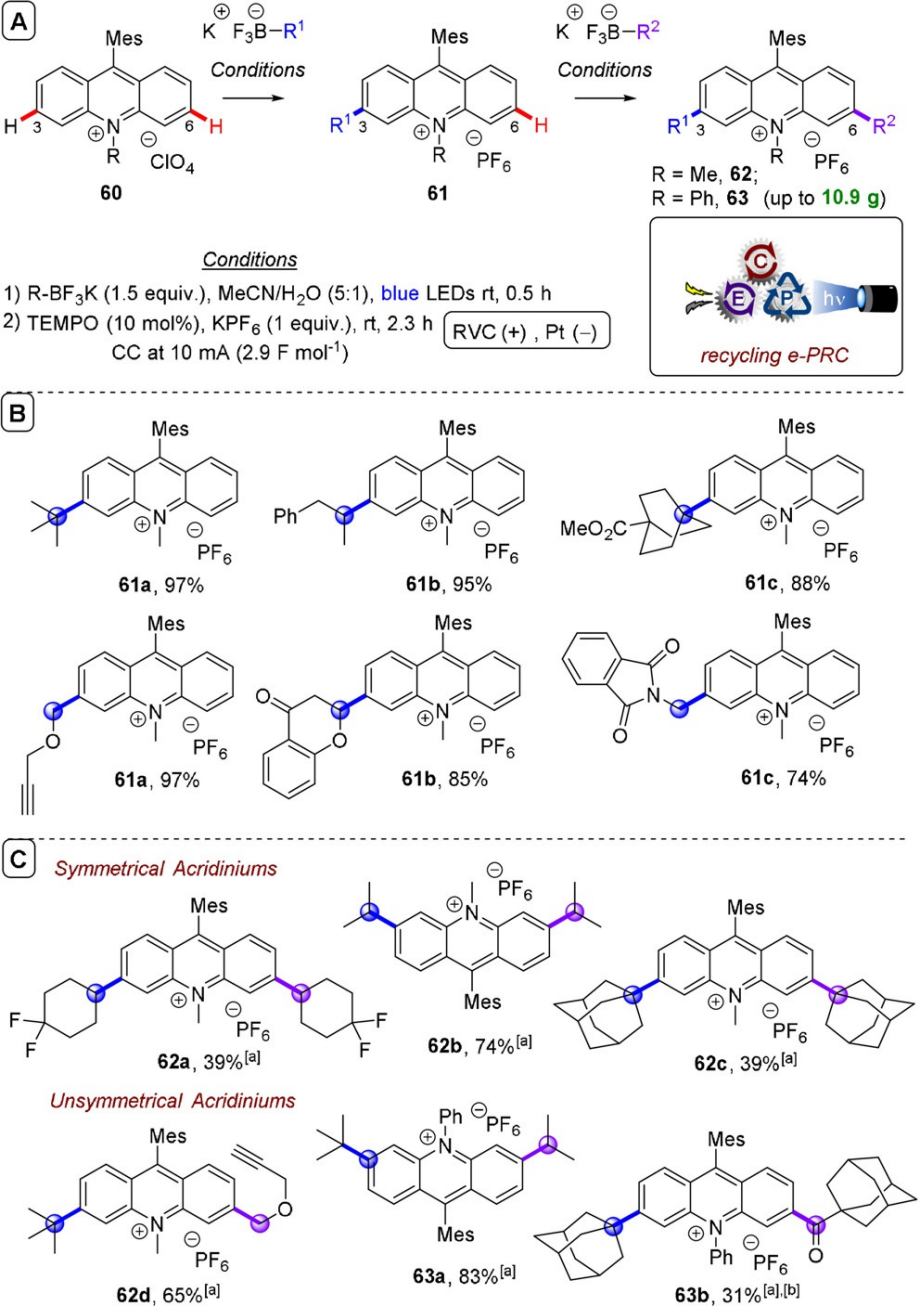
A) Iterative synthesis of 3,6‐disubstituted acridinium PRCats. B) Selected scope of monosubstituted products. C) Selected scope of disubstituted products. [a] Overall yield after both steps. [b] A 1,4‐dihydropyridine‐based alkyl donor was used.

**Figure 29:**
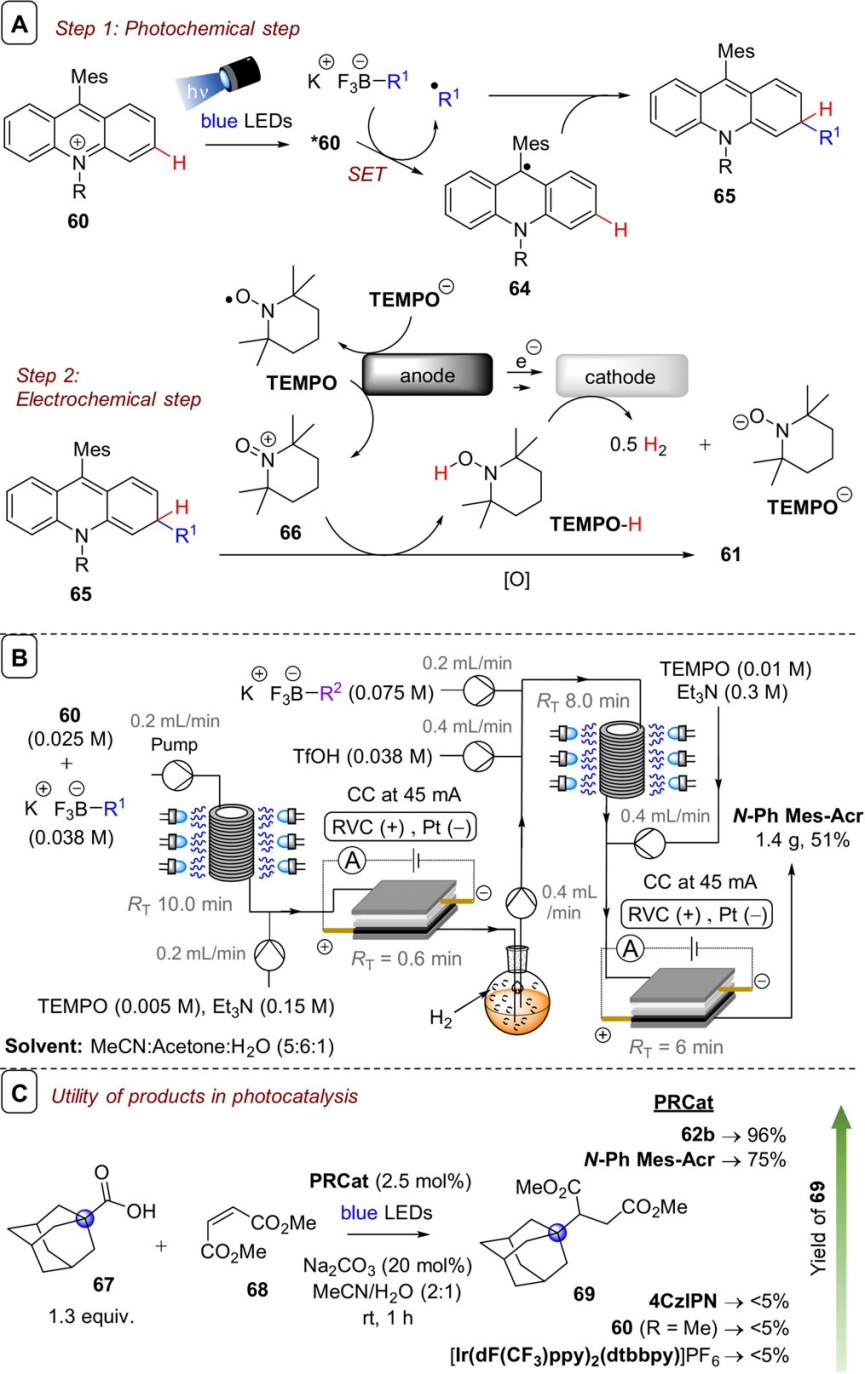
A) Proposed mechanism for each step. B) End‐to‐end continuous flow photoelectrochemical synthesis of functionalized acridinium salts. C) Utility of 3,6‐disubstituted acridinium salts in photocatalysis.

**Scalable synthesis of acridinium salts**: Finally, recycling e‐PRC was used by Xu's group to synthesize a library of 3/6‐substituted acridinium PRCats from an acridinium core **60** using trifluoroborate salts (Figure 28 A).[^57d^, ^57e^] Reactions were conducted iteratively to afford either monosubstituted PRCats **61** in very good to excellent (74–97 %) yields in one step (Figure 28 B), or disubstituted PRCats **62**/**63** in modest to very good (31–83 %) yields over two steps (Figure 28 C). Impressively, different trifluoroborate salts can be employed at each step to furnish unsymmetrical 3,6‐disubstituted acridinium salts. In the photochemical step (step 1) of the proposed mechanism (Figure 29 A), photoexcitation of **60**, followed by SET reduction of *60 by the alkyl trifluoroborate salt, affords reduced form **64**. Addition of trifluoroborate‐derived alkyl radical to **64** affords **65**. In the electrochemical step (step 2), TEMPO is oxidized to **66** which engages **65** in oxidation, affording TEMPO‐H and radical **61**. TEMPO‐H undergoes reduction at the cathode to liberate H_2_ and TEMPO^−^, the latter of which is transformed back to TEMPO by SET at the anode. By passing a 0.05 M solution of **60** (R=Ph) through a flow photocoil into an electrochemical batch reactor for the first functionalization, then washing with KPF_6_ before repeating for the second functionalization (see reference for details), 10.9 g (80 % over both steps) of ***N*** **‐Ph Mes‐Acr** (structure in Figure 1) was successfully synthesized. In the first example of an end‐to‐end, semicontinuous homogeneous synthetic photoelectrochemical flow process, Xu and co‐workers transformed 2.0 g of **60** (R=Ph) into 1.4 g (51 % over both steps) of ***N*** **‐Ph Mes‐Acr** (Figure 29 B). Here, the authors found that Et_3_N was necessary to improve conversion in the electromediated dehydrogenation of **65**. However, Et_3_N was detrimental to the photochemical step, so bases were neutralized in situ by TfOH before the subsequent photocoil. A collection flask was required after the first electrochemical flow reactor in order to purge gas bubbles (H_2_).

To demonstrate the value of their 3,6‐disubstituted product acridinium salts (**62**), Xu and co‐workers compared a variety of established photocatalysts in the photocatalytic decarboxylative conjugate addition of **67** to **68** (Figure 29 C). While unsubstituted **Mes‐Acr** ^+^ (**60**, R=Me), an iridium photocatalyst and **4CzIPN** gave only trace products, the 3,6‐di‐*tert*‐butyl‐substituted acridinium salt (***N*** **‐Ph Mes‐Acr**) was effective and novel catalyst **62 b** gave a near‐quantitative yield of **69**. Interestingly, the nature of substituents at the 3,6‐positions was found to dramatically influence the lifetime. The lifetime of ***N*** **‐Ph Mes‐Acr** (T_1_) is ≈6.1 ns (Table 1), while the lifetime of **62 c** was much longer (*τ*=30.7 ns) and is the longest lifetime ever reported for an acridinium salt. Enhanced lifetimes may provide a rationale for the increased activity of acridinium PRCat derivatives synthesized herein.

**Future perspectives**: Recycling e‐PRC continues to pave the way to enhanced sustainability and safety in photocatalysis, by replacing sacrificial redox agents with cheap, non‐hazardous electrochemistry. In contrast to radical ion e‐PRC which typically uses 0.1 M of supporting electrolyte, a recurring theme in recycling e‐PRC is the ability to use lower electrolyte loadings, typically <1 to several equivalents, likely as a result of an undivided cell setup with shorter interelectrode distances. Applicability of the technology is clear: from the late‐stage functionalizations of pharmaceutically relevant molecules to the synthesis of novel acridinium scaffolds representing attractive functional materials and photocatalysts. Recent advances demonstrate how recycling e‐PRC in recirculated or semicontinuous flow systems enables scalability of reactions, at least to multigram scales. However, the use of recycling e‐PRC in the *reductive* direction is yet to be explored. Moreover, in most reports of oxidative recycling e‐PRC, hydrogen gas is evolved and is not utilized downstream. Harnessing the by‐product at the counter‐electrode within a synthetic transformation (for example, utilizing H_2_ in a subsequent catalytic hydrogenation,[^7b^, ^65^] isolating it, or conducting a paired electrolytic reaction^[66]^) will improve Faradaic efficiency and enhance sustainability further. In a general sense, the elimination of sacrificial cathodic or anodic processes in favour of a paired electrolytic system is deemed necessary to encourage uptake of electro‐ and photoelectrochemistry in process chemistry.^[67]^

## 3 Photoelectrochemical HAT Reactions

**Figure 30:**
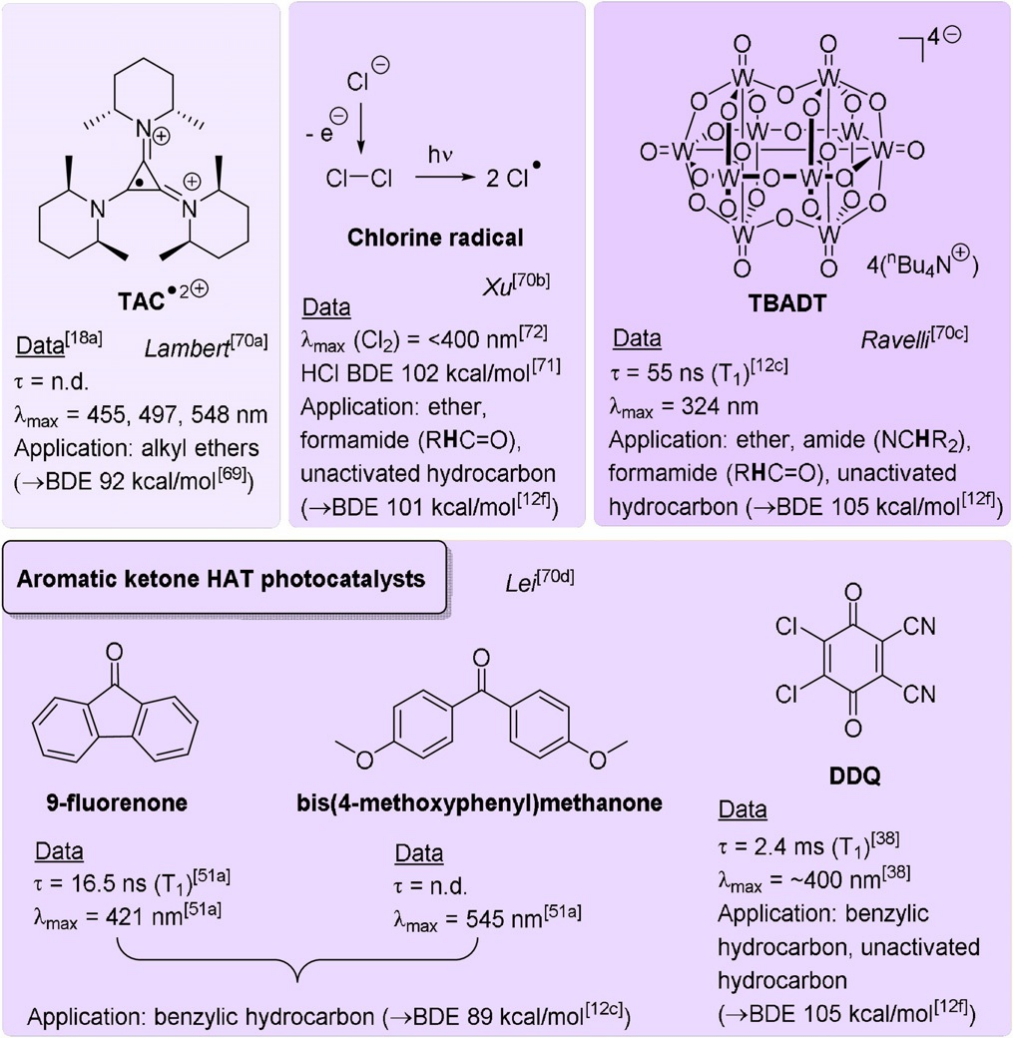
Photoelectrochemical HAT agents. Purple shading is indicative of C−H abstraction power. Selected photophysical and BDE data are shown.

By circumventing high redox potentials, HAT is an efficient way to generate radicals that are hard to obtain from direct photocatalytic SET redox activations or where high applied electrochemical potentials would be inappropriate.[^12^, ^68^] Examples covered in this section include C(sp^2^)–C(sp^3^) and C(sp^3^)–N couplings.^[70]^ Photoelectrochemical HAT agents and selected properties[^12c^, ^12f^, ^18a^, ^51a^, ^69^, ^70^, ^71^, ^72^] are displayed in Figure 30.

**Figure 31:**
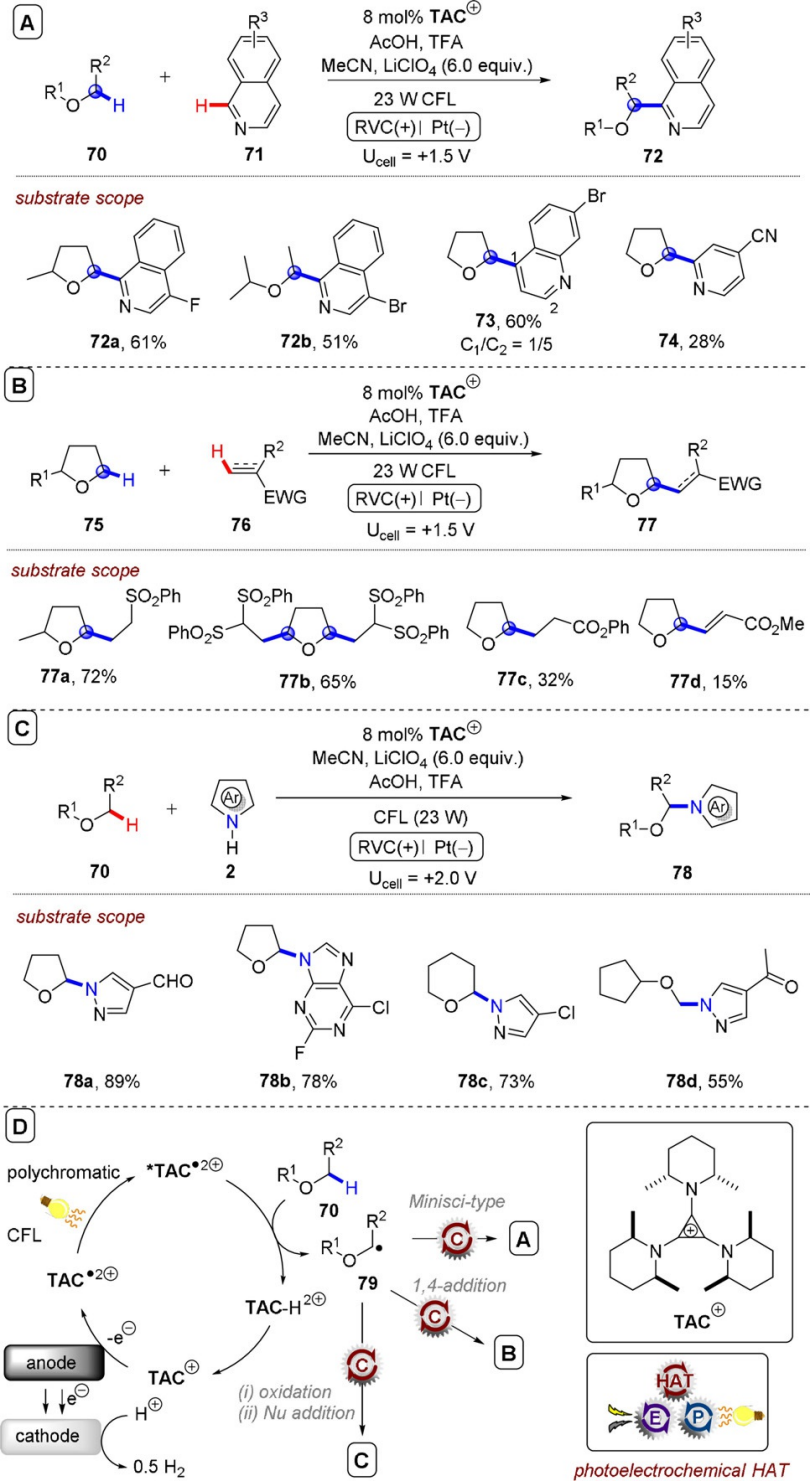
A) Photoelectrochemical HAT activation of alkyl ethers by a photoexcited cyclopropenium dication radical and participation of ethereal sp^3^ radicals in a Minisci‐type reaction. B) 1,4‐addition of ethereal sp^3^ radicals to electron‐deficient alkenes or alkynes. C) Oxidation of ethereal sp^3^ radicals and nucleophilic azolations. D) Proposed photoelectrochemical HAT mechanism.

Lambert and co‐workers reported a photoelectrochemical HAT activation of C(sp^3^)−H bonds of alkyl ethers (**70**, **75**).^[70a]^ Here, the trisaminocyclopropenium ion (**TAC** ^+^) developed by the group for radical ion e‐PRC super‐oxidations was employed as a catalyst. Upon anodic oxidation of **TAC** ^+^ and photoexcitation of **TAC^.2+^**, the authors proposed that the excited radical dication *TAC^.2+^ engaged C(sp^3^)−H bonds of ethers in HAT to afford sp^3^ radical **79** (Figure 31 D), which either 1) engaged in a Minisci‐type reaction with quinoline derivatives like **71** to afford products **72**–**74** (Figure 31 A); 2) underwent 1,4‐addition to electron‐deficient alkenes or alkynes **76** to afford products **77 a**–**d** (Figure 31 B); or 3) underwent further oxidation to an oxocarbenium ion and then nucleophilic azolation with *N*‐heteroarenes **2** to afford products **78 a**–**d** (Figure 31 C). Yields were generally satisfactory to excellent for all downstream transformations (31–89 %). Following the proposed HAT step, **TAC**‐H^2+^ releases a proton which is reduced to hydrogen at the cathode.

The observation of a kinetic isotopic effect (KIE, *k*_H/D_=3.0) confirmed rate‐determining C−H cleavage. Despite the apparent bulkiness of *TAC^.2+^ as a HAT agent, the authors proposed that high selectivity for secondary C(sp^3^)–H> tertiary C(sp^3^)–H corroborates HAT as the mechanism. However, the lifetime of *TAC^.2+^ is unknown, and as a doublet excited state, it is likely ultrashort‐lived such that HAT might require preassembly of ethers and **TAC^.2+^**.

**Figure 32:**
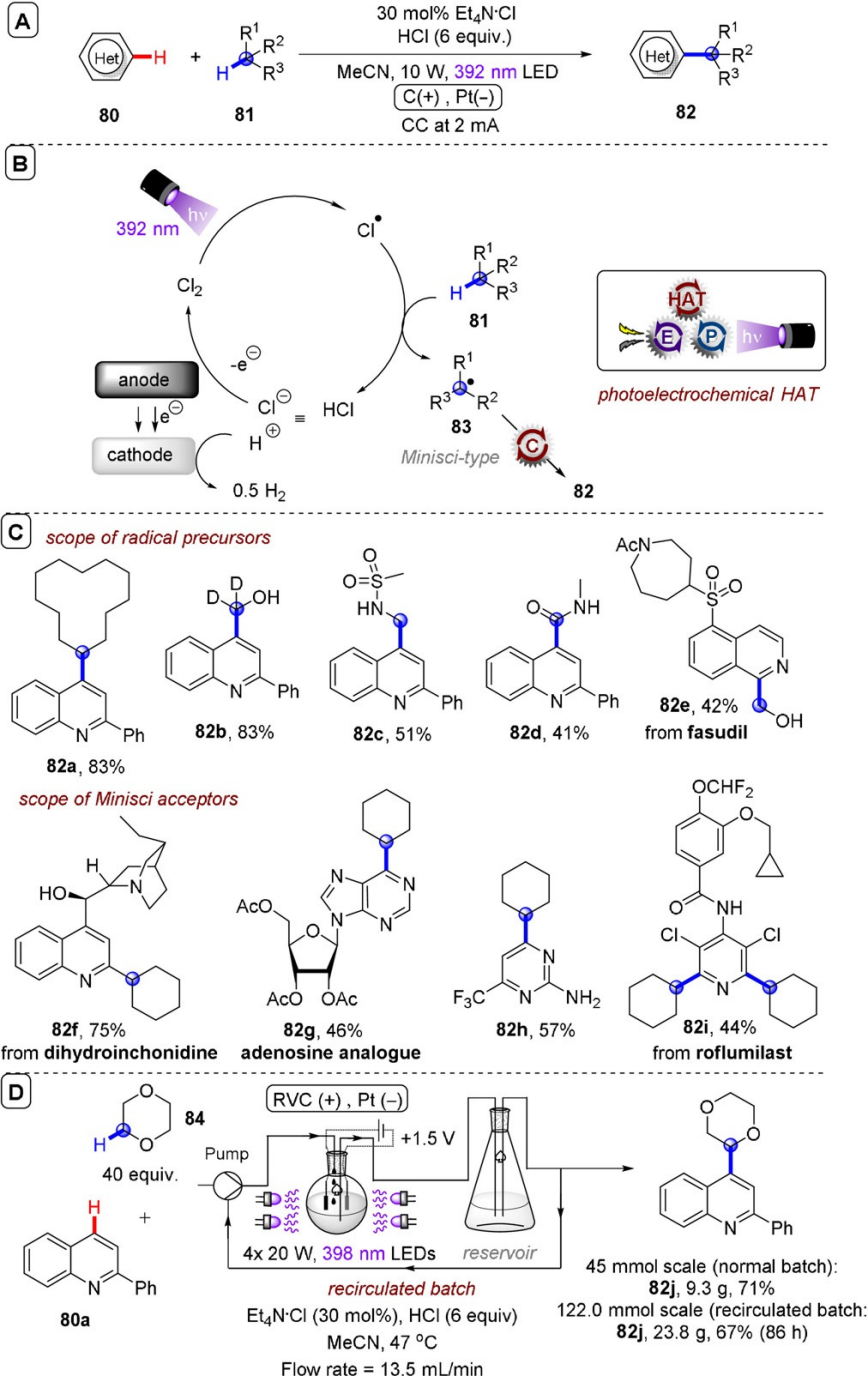
A) Photoelectrochemically generated chlorine radicals in C(sp^3^)–H HAT activation for a Minisci‐type reaction; B) Proposed mechanism. C) Selected examples. D) Recirculated flow photoelectrochemical HAT activation of 1,4‐dioxane and Minisci‐type reaction.

The Xu group reported an elegant photoelectrochemical pathway to activate C(sp^3^)−H bonds of substrates (**81**) using chlorine radicals as HAT agents generated in situ within an undivided cell.^[70b]^ The generated C(sp^3^) radical (**83**) again participated in Minisci‐type reaction with heteroarenes (**80**) (Figure 32 A). In their proposed mechanism, anodic oxidation of Cl^−^ to Cl_2_ first occurs. Subsequent light irradiation leads to homolysis of Cl_2_,^[73]^ generating Cl**^.^** as a powerful HAT agent. Continuous in situ generation of Cl_2_ by anodic oxidation and its consumption in the reaction avoids the direct use of toxic Cl_2_ gas (Figure 32 B). Since the bond dissociation enthalpy (BDE) of HCl is high (102 kcal mol^−1^),^[71]^ Cl**^.^** is a powerful HAT agent that successfully engages various C(sp^3^)−H bonds in a thermodynamically favoured process. As well as ethers, radicals were successfully accessed from C(sp^2^)–H positions of formamides and even C(sp^3^)–H positions of hydrocarbons were successfully engaged. The substrate scope with respect to both radical precursors and heteroarenes was relatively broad, tolerating many sensitive functional groups and affording products like **82 a**–**f** generally in modest to excellent (40–94 %) yields (Figure 32 C). The ability to replace photo‐ or electroactivated photocatalysts (**TBADT**, **TAC^+^**) with Cl**^.^** from HCl readily available in all laboratories is a key advantage of this method. Without noticeable erosion in the yield of **82 f**, a batch reaction was done on a 45 mmol scale. Even a 122 mmol batch reaction was achieved by recirculating the reaction mixture through a reservoir (Figure 32 D). As per previous Minisci‐type photoelectrochemical reactions (Section 2), late‐stage functionalizations were achieved for complex and bioactive molecules such as dihydroinchonidine, fasudil, roflumilast and even an adenosine analogue (Figure 32 C). While this process involved the photoelectrochemical generation of chlorine radicals, it is worth noting that the photoelectrochemical generation of iodine radicals for HAT at benzylic positions was reported earlier by Stahl and co‐workers,^[74]^ and was reviewed previously.^[9]^

**Figure 33:**
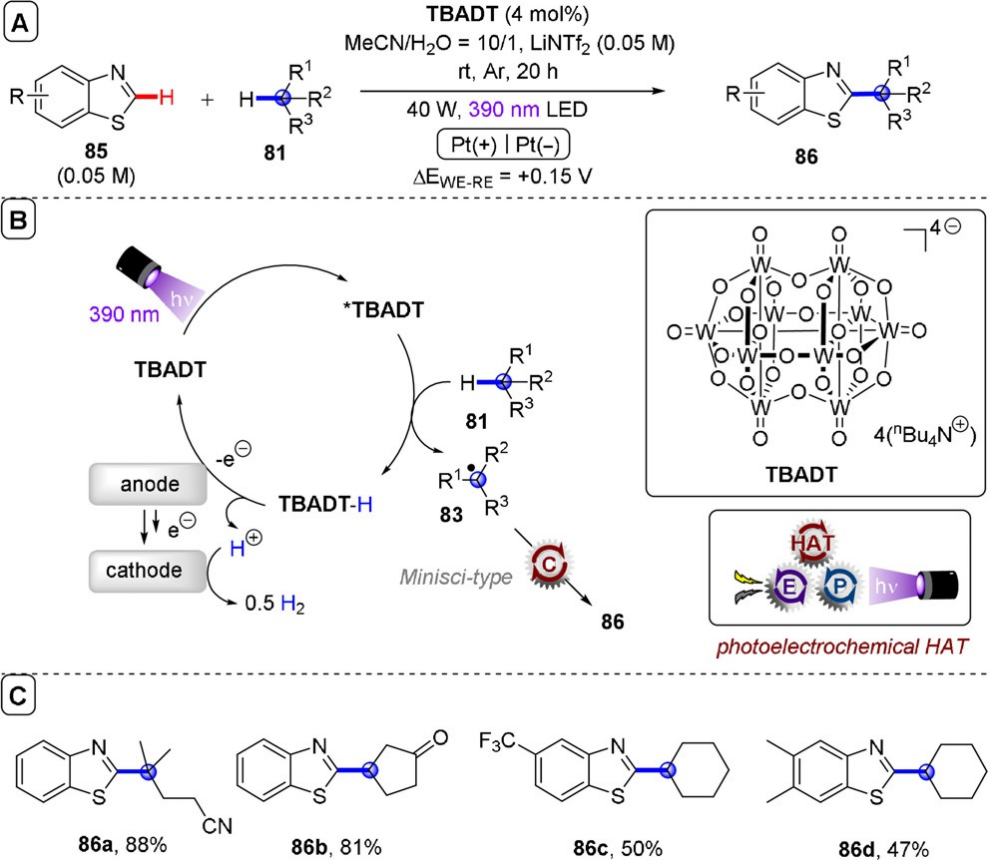
A) Photoelectrochemical HAT involving electro‐recycling of **TBADT** ((^n^Bu_4_N)_4_[W_10_O_32_]). B) Proposed mechanism. C) Selected examples.

It is well established that the photoexcited state of tetra‐*n*‐butylammonium decatungstate, **TBADT** ((^n^Bu_4_N)_4_[W_10_O_32_]) is one of the most powerful HAT agents reported. It engages challenging C(sp^3^)−H bonds in HAT, including alkyl ethers, alkyl nitriles and even light hydrocarbons like methane.[^12a^, ^12c^, ^12f^] Ravelli and co‐workers recently demonstrated electro‐recycling of **TBADT** in a photoelectrochemical HAT process (Figure 33 A).^[70c]^ Photoexcited **TBADT** engaged the remote C(sp^3^)−H bonds of nitriles and carbonyls as well as hydrocarbons in HAT to afford C(sp^3^) radicals and **TBADT**‐H. Benzothiazoles (**85**) were explored as the C(sp^3^) radical trapping partner, affording coupling products like **86 a**–**d** (Figure 33 C) in moderate to excellent (47–88 %) yields. **TBADT** is regenerated from **TBADT**‐H by anodic oxidation at a very low applied potential (Δ*E*_WE‐RE_=+0.15 V) in a divided cell (Figure 33 B), such that the reaction could even be driven successfully by two AAA batteries. Laser flash photolysis confirmed the quenching of ^**3**^ TBADT* by **81** and by a dihydro‐precursor of **86**, but not by the LiNTf_2_ electrolyte.

**Figure 34:**
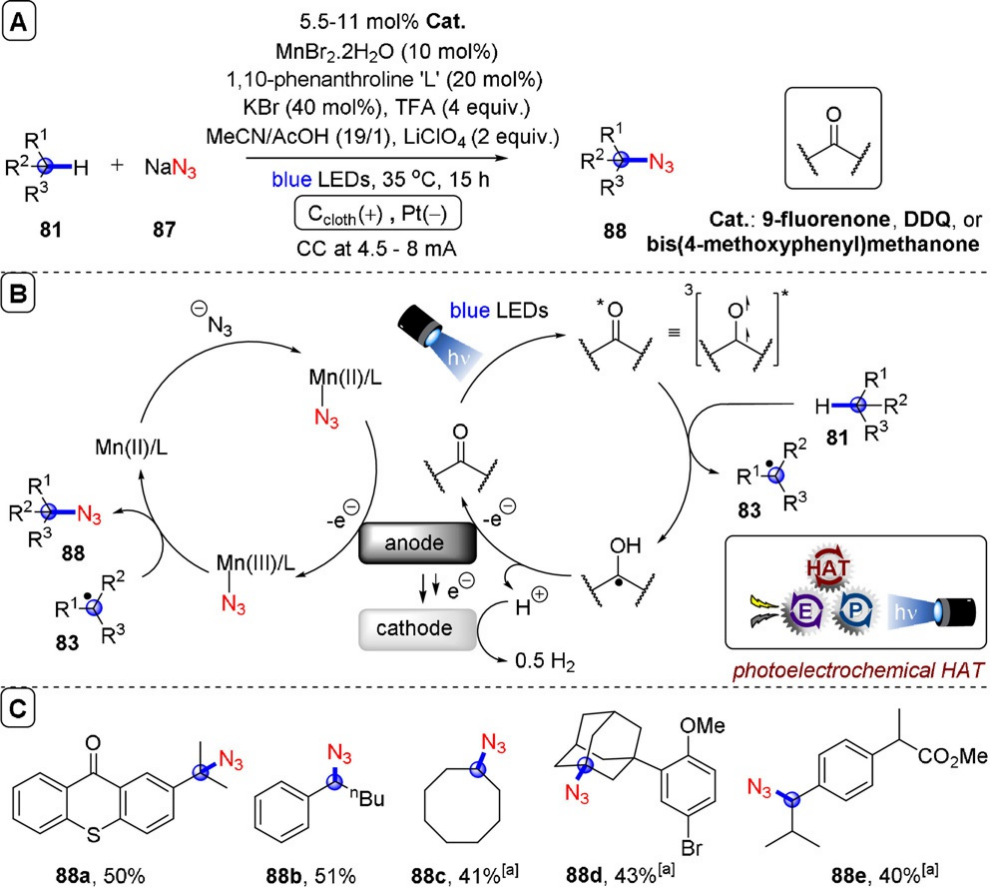
A) Photoelectrochemical HAT azidation of C(sp^3^)−H bonds. B) Proposed mechanism. C) Selected examples. [a]  **Cat**.=**DDQ**.

Lei and co‐workers group reported a photoelectrochemical oxidative azidation of C(sp^3^)−H bonds of substrates (**81**) in an undivided cell, in which electrochemistry plays multipole roles (Figure 34 A).^[70d]^ Upon photoexcitation of an aromatic ketone catalyst (**Cat**.) and intersystem crossing, the long‐lived triplet excited state abstracts a C(sp^3^)–H atom from either activated benzylic positions (when **Cat**.=9‐fluorenone or bis(4‐methoxyphenylmethanone) or unactivated hydrocarbons (when **Cat**.=**DDQ**), affording an C(sp^3^) radical **83** which reacts with a Mn^III^‐azide complex species to form the azidated product **88**. This Mn^III^‐azide complex is electrogenerated in situ from its precursor Mn^II^‐azide complex. The authors proposed that, upon photoexcited HAT, reoxidation back to the carbonyl is simultaneously mediated by anodic oxidation (Figure 34 B), while liberated protons are reduced to hydrogen at the cathode. Benzylic and unactivated hydrocarbons were azidated to afford a broad scope of products such as **88 a**–**e** in modest to excellent (31–99 %) yields (Figure 34 C). Reactivities and selectivities were generally higher for tertiary benzylic C(sp^3^)–H azidation compared to secondary benzylic azidations. The azidation of a cumene derivative was successfully achieved on gram scale after extended reaction time (71 % yield after 72 h). Late‐stage azidation of bioactive molecules such as a differin precursor and ibuprofen methyl ester (affording **88 d,e**) was accomplished when **DDQ** was used as a catalyst.

In their mechanistic studies, the authors found by cyclic voltammetry that the oxidation of NaN_3_ occurred at a lower potential than substrates and photocatalysts. This led them to suggest the azide radical was preferentially formed under the anodic conditions of the reaction. TEMPO was found to inhibit the reaction and by EPR the authors were able to detect the azide radical. In their optimization, authors found that the reaction did proceed appreciably in the dark without catalyst (45 % yield), although the rate of reaction was markedly accelerated by irradiation (61 % yield). To rationalize progress of the reaction in the dark, they suggested that the azide radical can also engage in HAT.

## 4 Reactor Platforms

**Figure 35:**
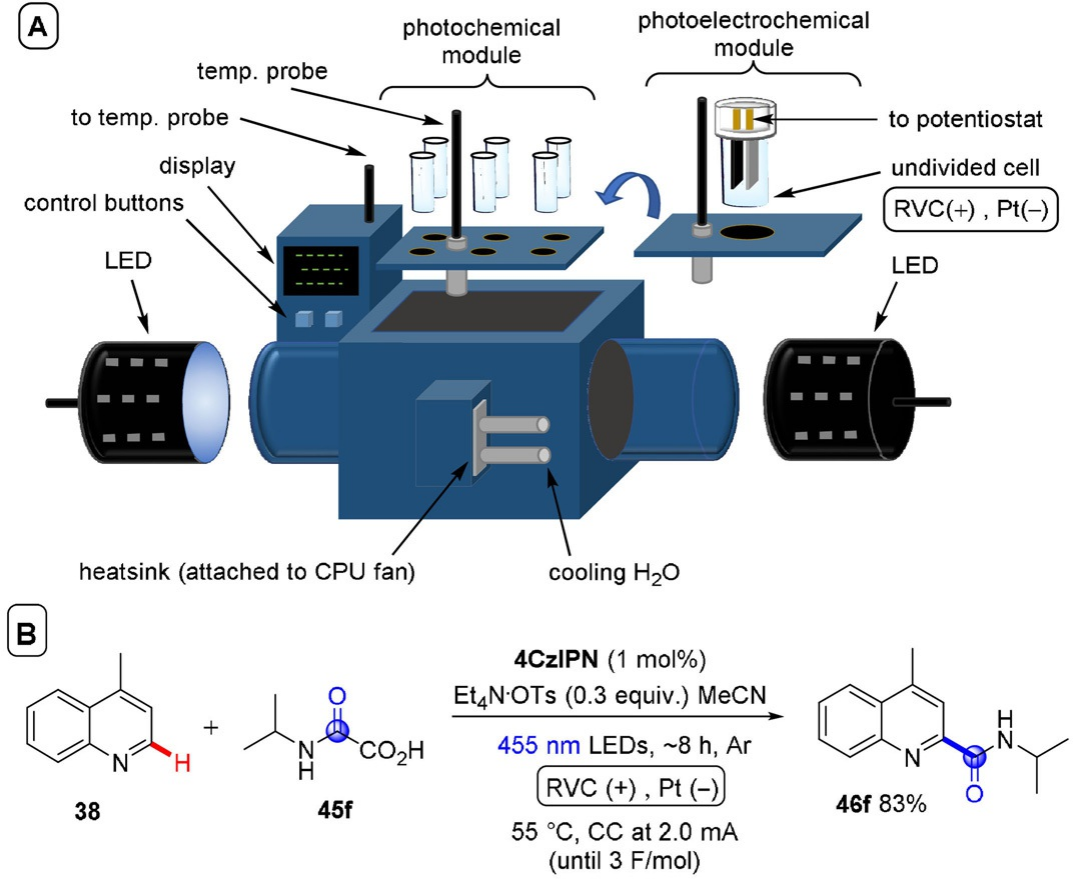
A) A 3D‐printed reactor for photochemical and photoelectrochemical reactions. B) Application to recycling e‐PRC carbamoylation of heteroarenes.

Of key importance to the discovery of new PEC reactions and their uptake in industry is the requirement for robust reactor platforms that deliver reproducible chemistry. To this end, a 3D‐printed photoreactor accommodating two interchangeable high‐power commercial LED lamps and up to six vials was reported by Schiel and co‐workers at Boehringer Ingelheim (Figure 35 A).^[75]^ The reactor gave excellent reproducibility, both vial‐to‐vial and of literature PRC reaction yields. By exchanging the vial holder module, the reactor accommodated an undivided cell photoelectrochemical reactor vial driven externally by a benchtop commercial potentiostat.^[76]^ The recycling e‐PRC heteroarene carbamoylation^[57b]^ was successfully reproduced, affording **46 f** in 83 % yield (Figure 34 B). Since 3D printing has previously converted a commercial benchtop potentiostat into a compact flow electrochemical reactor,^[77]^ it is likely that commercial 3D‐printed photoelectrochemical batch and flow systems will soon be available to practitioners, both in divided and undivided modes.

**Figure 36:**
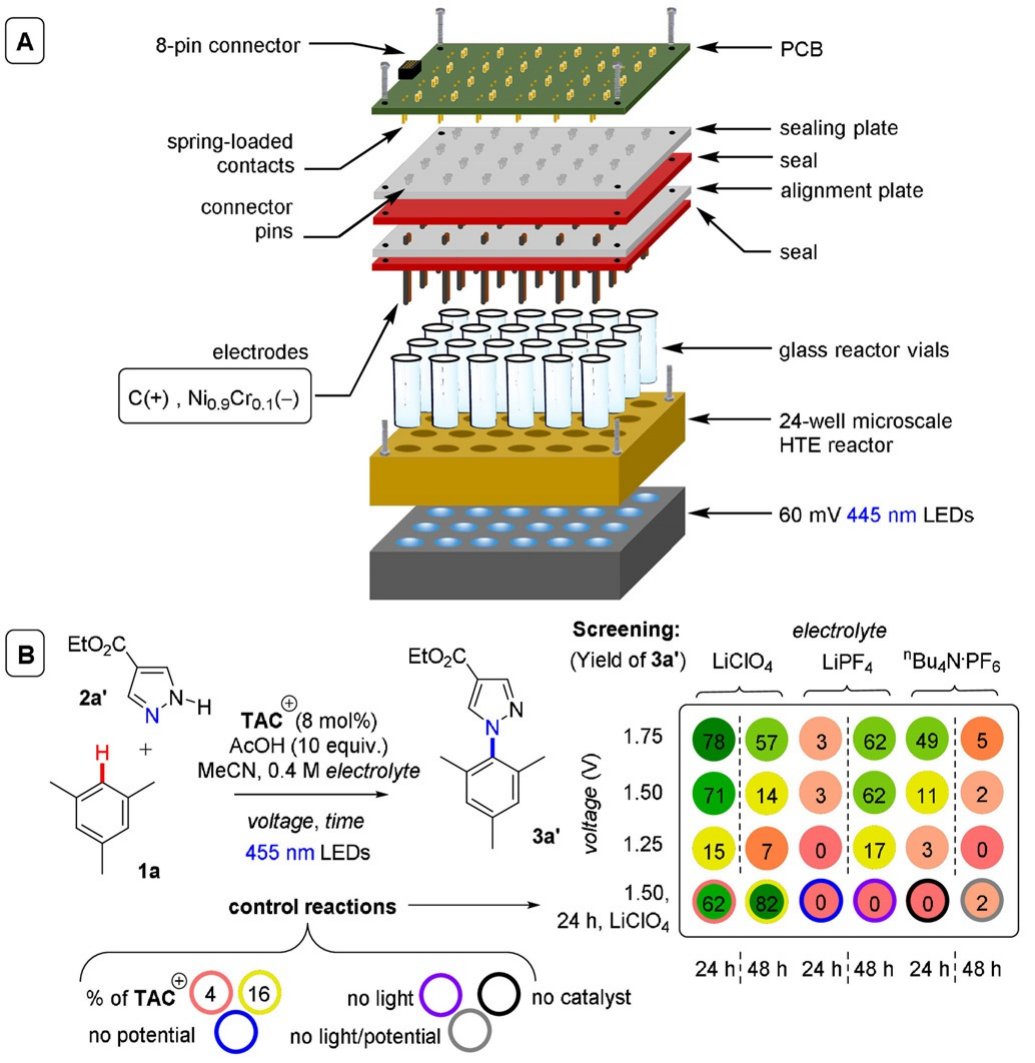
A) A compact, high‐throughput screening platform for photoelectrochemistry. B) Results of screening a radical ion e‐PRC reaction.

A key challenge in the discovery of molecular photoelectrochemical reactions is the number of variables that arise when combining SOE with PRC. Light wavelength, light intensity, current, potential, electrode materials, divided vs. undivided cell, temperature, catalyst choice and loading, electrolyte choice and loading all influence the reaction outcome. The critical influence of light intensity and reaction temperature in PRC has been recently highlighted.^[78]^ High‐throughput screening is a powerful tool for reaction discovery, allowing multiple variables to be simultaneously explored.^[79]^ Lin, Lehnherr, Kalyani and co‐workers developed a compact, high‐throughput microscale electrochemical reactor that was successfully applied to screen up to 24 conditions at once in a radical ion e‐PRC reaction under constant potential (Figure 36), including control reactions.^[80]^ The reactor increased the reaction rate threefold, likely due to increased transmission of light on the microscale and LED optical power. Vial‐to‐vial reproducibility was high (identical reactions with an average 75 % yield gave a 5 % standard deviation) confirming the robustness of the system for discovery.

## 5 Summary and Outlook

Synthetic photoelectrochemistry (PEC) involving in situ generated homogeneous photocatalysts is a rapidly growing research frontier in single‐electron transfer chemistry and organic synthesis. Radical ion electrochemically mediated photoredox catalysis (e‐PRC) pushes the boundaries of the redox window further than ever before, achieving unprecedented oxidations and reductions in a controlled, selective manner that can be leveraged to construct, or cleave, strong bonds. While controversy continues to surround the operating mechanisms of reactions proposed to involve radical ion photocatalysts, substrate–catalyst preassembly provides a convincing interpretation of reactivity patterns and offers exciting new opportunities for selectivity control that challenge conventional parameters like the thermodynamic redox potential. Recycling electrochemically mediated photoredox catalysis forges ahead of conventional photoredox catalysis in improving sustainability, scalability, safety and cost‐efficiency of reaction conditions, with sacrificial chemical redox additives being substituted for cheap, benign electricity. Both subcategories of e‐PRC leverage the general selectivity benefits of substrate engagement with a photoexcited state in bulk solution, mitigating against over‐reduction or over‐oxidation processes and harnessing reactive intermediates that may normally undergo further redox chemistry at electrode surfaces or lead to grafting/passivation. The combination of PEC and HAT opens new opportunities for synthesis, including new photoexcited HAT agents or electro‐recycling established ones, as well as providing new access to ground‐state HAT agents from inexpensive, abundant precursors. Finally, in addition to promising initial efforts in the scale‐up of these chemistries in recirculated or continuous flow, new reactor platforms designed for high reproducibility, control of reaction variables and high‐throughput experimentation pave the way to photoelectrochemistry becoming a tool accessible to both academic and industrial chemists alike.

## Conflict of interest

The authors declare no conflict of interest.

## Biographical Information

*Shangze Wu was born in Fushun (P.R. China). He received his Ph.D. in 2017 under the supervision of Prof. Shengming Ma at Zhejiang University (China), where he investigated allene chemistry and C−H bond activation. From 2017–2020, he worked as a postdoctoral researcher exploring titanium‐mediated epoxide chemistry with Prof. Andreas Gansäuer at the University of Bonn (Germany). In 2020, he joined the group of Dr. Joshua P. Barham for postdoctoral research on synthetic photoelectrochemistry*.

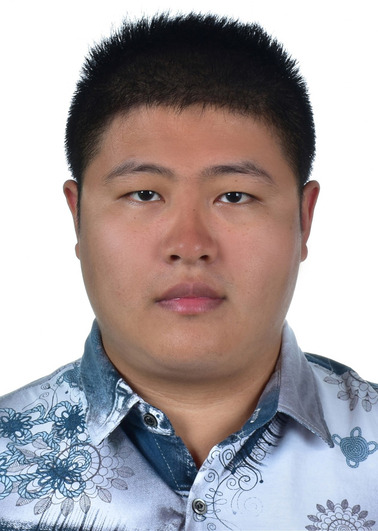

## Biographical Information

*Jaspreet Kaur was born in India. She received her B.Sc. in 2017 from the University of Brighton (UK), and her M.Sc. in 2020 from the Elite Network of Bavaria's Synthesis and Catalysis Master Programme under the supervision of Dr. Joshua P. Barham at the University of Regensburg (Germany). In her Ph.D. studies she is focussing on photocatalysis and photoelectrochemistry in organic synthesis*.

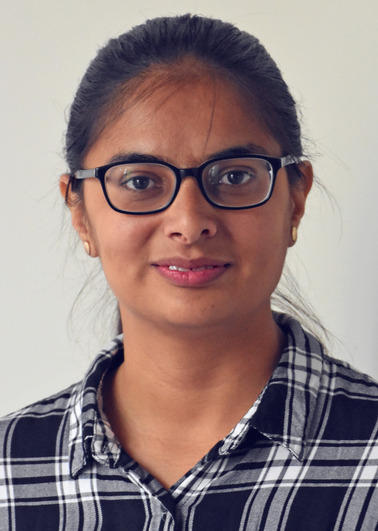

## Biographical Information

*Tobias A. Karl was born in Neumarkt (Opf., Germany). He received his B.Sc. in 2016 from the Technical University of Nürnberg, and his M.Sc. in 2018 from the University of Regensburg under the supervision of Prof. Burkhard König. As a Ph.D. student at the University of Regensburg and supported by the Deutsche Bundesstiftung Umwelt (DBU), he focuses on photo‐, electro‐ and photoelectrochemistry in organic synthesis*.

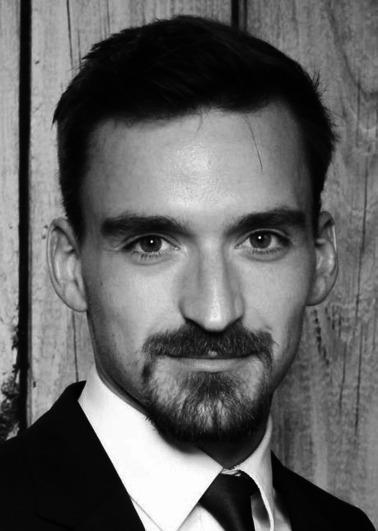

## Biographical Information

*Xianhai Tian was born in Xinyang (P.R. China). He received his Ph.D. in 2019 under the supervision of Prof. A. Stephen K. Hashmi at the University of Heidelberg (Germany), where he investigated gold carbene and nitrene chemistry. In 2020, he joined the group of Dr. Joshua P. Barham for postdoctoral research on synthetic photoelectrochemistry*.

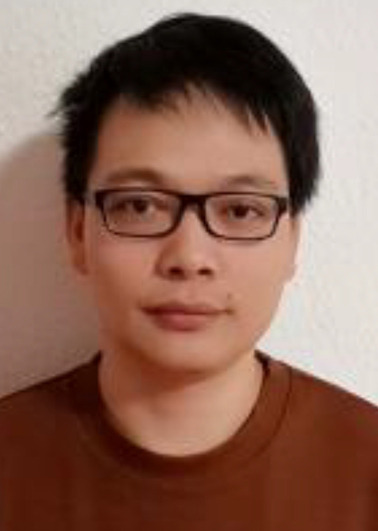

## Biographical Information

*Joshua P. Barham was born in Watford (UK). He received his industry‐based Ph.D. in 2017 under the supervision of Prof. John A. Murphy and Dr. Matthew P. John at the University of Strathclyde and GSK (UK). His postdoctoral studies with Prof. Yasuo Norikane and Prof. Yoshitaka Hamashima at AIST and the University of Shizuoka (Japan) specialized in flow chemistry and photoredox catalysis. Since 2019 his group has investigated photo‐, electro‐, photoelectro‐ and continuous flow organic synthesis at the University of Regensburg, supported by a Sofja Kovalevskaja Award*.